# Cross-sectional imaging of acute gynaecologic disorders: CT and MRI findings with differential diagnosis—part I: corpus luteum and haemorrhagic ovarian cysts, genital causes of haemoperitoneum and adnexal torsion

**DOI:** 10.1186/s13244-019-0808-5

**Published:** 2019-12-19

**Authors:** Massimo Tonolini, Pietro Valerio Foti, Valeria Costanzo, Luca Mammino, Stefano Palmucci, Antonio Cianci, Giovanni Carlo Ettorre, Antonio Basile

**Affiliations:** 1 Department of Radiology, “Luigi Sacco” University Hospital, Via G.B. Grassi 74, 20157 Milan, Italy; 2 Department of Medical Surgical Sciences and Advanced Technologies, Radiology I Unit, University Hospital “Policlinico-Vittorio Emanuele”, Via Santa Sofia 78, 95123 Catania, Italy; 3 Department of General Surgery and Medical-Surgical Specialties, Institute of Obstetrics and Ginecology, University of Catania, Catania, Italy

**Keywords:** Gynaecologic emergencies, Corpus luteum, Ectopic pregnancy, Computed tomography, Magnetic resonance imaging

## Abstract

Acute gynaecologic disorders are commonly encountered in daily clinical practice of emergency departments (ED) and predominantly occur in reproductive-age women. Since clinical presentation may be nonspecific and physical findings are often inconclusive, imaging is required for a timely and accurate diagnosis. Although ultrasound is the ideal non-invasive first-line technique, nowadays multidetector computed tomography (CT) is extensively used in the ED, particularly when a non-gynaecologic disorder is suspected and differential diagnosis from gastrointestinal and urologic diseases is needed. As a result, CT often provides the first diagnosis of female genital emergencies. If clinical conditions and scanner availability permit, magnetic resonance imaging (MRI) is superior to CT for further characterisation of gynaecologic abnormalities, due to the excellent soft-tissue contrast, intrinsic multiplanar capabilities and lack of ionising radiation.

The purpose of this pictorial review is to provide radiologists with a thorough familiarity with gynaecologic emergencies by illustrating their cross-sectional imaging appearances. The present first section will review the CT and MRI findings of corpus luteum and haemorrhagic ovarian cysts, gynaecologic haemoperitoneum (from either ruptured corpus luteum or ectopic pregnancy) and adnexal torsion, with an emphasis on differential diagnosis. Additionally, comprehensive and time-efficient MRI acquisition protocols are provided.

## Teaching points

- The CT and MRI hallmark of a corpus luteum is a cystic adnexal structure with crenulated, strongly enhancing periphery
- Key differential diagnoses of haemorrhagic ovarian cysts include endometrioma, dermoids and mucinous cystic tumours
- Gynaecologic haemoperitoneum with or without active bleeding is most usually caused by ruptured corpus luteum
- Pelvic haemoperitoneum, partly haemorrhagic adnexal mass and positive β-hCG test allow diagnosing ruptured ectopic pregnancy
- A benign ovarian mass (most usually a mature cystic teratoma) underlies over half of adnexal torsion cases

## Introduction

Although less prevalent than obstetric issues, acute gynaecologic disorders in non-pregnant women are not uncommon in busy emergency departments (ED). Ultrasound (US) represents the ideal non-invasive technique for initial investigation of suspected genital diseases. However, due to fast acquisition and widespread availability on a 24/7 basis, multidetector computed tomography (CT) now represents the ‘workhorse’ imaging modality in the ED and quickly provides accurate and reproducible diagnosis of most acute abdominal and pelvic complaints. As a result, acute abnormalities of the female genital organs are increasingly detected on urgent CT studies, most usually when clinical manifestations are non-specific and a gynaecologic disorder is not prospectively considered as the primary origin of pelvic pain, or when differential diagnosis from gastrointestinal and urologic conditions (e.g. acute appendicitis, diverticulitis, pyelonephritis and renal colic) is required. Furthermore, CT provides the first diagnosis of an acute gynaecologic disorder when transvaginal US (in our country, mostly performed by gynaecologists) is unavailable or inconclusive, and when genital abnormalities extend beyond the sonographic field of view or require further characterisation. In these situations, the role of the radiologist is to alert ED physicians about the possible or probable presence of an unsuspected genital disease that warrants immediate gynaecologic consultation [1–4].

Magnetic resonance imaging (MRI) represents the ideal modality for characterisation of abnormal or inconclusive sonographic and CT findings. Advantages of MRI over CT include the lack of radiation exposure, native multiplanar acquisition, optimal soft-tissue contrast and tissue characterisation including the possibility of demonstrating the presence of fat and blood products. Although limited by scanner availability, exam duration and cost, the use of MRI in urgent settings has steadily grown over the last decade, particularly in paediatric patients and child-bearing age women, to avoid the risks of administering ionising radiation to the genital organs [5–7].

This pictorial essay aims to provide radiologists with an increased familiarity with recognition and characterisation of acute gynaecologic disorders on CT and MRI, in order to allow timely diagnosis and appropriate treatment. The present instalment reviews the cross-sectional imaging appearances of corpus luteum and haemorrhagic ovarian cysts, gynaecologic haemoperitoneum (from either ruptured corpus luteum or ectopic pregnancy) and adnexal torsion, with an emphasis on differential diagnosis. In the second section, uterine emergencies and the spectrum of pelvic inflammatory disease (PID) will be presented [8, 9].

## Cross-sectional imaging techniques

### Multidetector CT acquisition technique

To investigate patients with acute abdominal or pelvic pain, intravenous iodinated contrast agent is generally used, unless contraindicated by allergy or impaired renal function. Alternatively, gynaecologic abnormalities (such as adnexal enlargement) may be encountered in unenhanced abdominal CT studies, such as those requested to investigate suspected urolithiasis and acute renal colic: in these situations, further investigation with contrast-enhanced CT or better MRI should be suggested after gynaecologic consultation [2, 4, 8].

In postmenopausal women, most institutions perform a preliminary unenhanced acquisition followed by optional arterial and mandatory portal venous phase CT scanning. In patients with hip prosthesis or other metallic implants, metal artefact reduction algorithms decrease beam-hardening effects that impair the assessment of the pelvic organs [10].

In reproductive age women with unspecific abdominal complaints, a limited CT protocol that includes a single acquisition in the portal venous phase is beneficial to limit the ionising radiation dose. If available, novel CT techniques such as iterative reconstruction should be used. When a gynaecologic disease is prospectively considered or sonography shows a non-trivial pelvic effusion, we suggest obtaining also a precontrast scanning at least of the pelvis, as it may be helpful to discriminate high attenuation reflecting the presence of blood or debris within a cyst or collection from contrast enhancement. Regardless of patient age, an additional arterial-phase (CT-angiography) acquisition using bolus tracking technique with the region-of-interest in the abdominal aorta is warranted when a concern for blood loss exists, such as in patients with vaginal bloody discharge, dropping haematocrit, hypotension or haemodynamic instability and after sonographic detection of haemoperitoneum [2, 4, 8].

### CT interpretation

In state-of-the art multidetector scanners, CT generates almost isotropic sub-millimetre voxels that allow reconstruction of high-resolution images along arbitrary planes. Even if a gynaecologic disease is not prospectively suspected, in females, the routine reconstruction of 3- to 5-mm-thick sagittal and coronal CT images of the pelvis is recommended to depict the anatomy of normal and diseased genital organs. Sagittal viewing allows appropriate assessment of size, morphology and enhancement patterns of the uterus, the latter markedly varies according to age, menstrual phase and delay between contrast injection and CT acquisition. Additional MRI-like oblique-axial and oblique-coronal reconstructions (perpendicular and parallel to the main uterine axis, respectively) may be helpful to discriminate extra-uterine structures and to better assess topography of uterine abnormalities such as subserosal or pedunculated leiomyomas [1–4, 11].

At CT, the fallopian tubes are generally not visible in normal conditions but become identifiable as tubular structures when dilated by intraluminal fluid, secretions or blood. The normal ovaries measure approximately 3 × 3 × 2 cm and show low attenuation with a faintly recognizable follicular structure on contrast-enhanced images. Identification of the ovaries relies on their location close to the external iliac vessels, posterior to the round ligament. Recognition of the suspensory ligament (which connects the ovary to the pelvic sidewall) as a linear or fan-shaped soft-tissue band allows diagnosis of ovarian versus non-ovarian masses. The ovaries are located anterior or antero-medial to the pelvic ureters, conversely iliac lymph nodes lie lateral or posterolaterally [3, 12].

### MRI acquisition technique

Our comprehensive MRI acquisition protocol of the pelvis for acute pelvic pain or suspected acute gynaecologic disorders is performed on a closed-configuration 1.5-Tesla superconducting MRI scanner (Signa HDxT; GE Healthcare, Milwaukee, Wisconsin, USA) using an eight-channel high-resolution phased-array torso coil with array spatial sensitivity technique (ASSET) parallel acquisition, with the patient lying in the supine position (entry position feet first). An antispasmodic agent such as glucagon or N-butyl-scopolamine, unless contraindicated (e.g. unstable cardiac disease, tachycardia, diabetes, acute angle-closure glaucoma and phaeochromocytoma) can be administered intravenously or intramuscularly to reduce bowel motion artefacts [13, 14].

This state-of-the-art MRI protocol (Table 1) relies on T2- and T1-weighted sequences with and without fat suppression and diffusion-weighted imaging (DWI) sequences. Multiplanar T2-weighted sequences are crucial in the assessment of the female pelvis since they provide excellent soft-tissue contrast of the genital organs and surrounding structures. Fat-suppressed T2-weighted images increase the conspicuity of inflammatory lesions and are useful to identify ascites, complex fluid, oedema of tissues and perivisceral fat [15]. Optional gradient-echo T2*-weighted sequences are suitable to detect haemorrhage and air bubbles due to their sensitivity to magnetic susceptibility effects but are particularly prone to susceptibility artefacts from bowel gas. Precontrast fat-suppressed T1-weighted sequences allow differentiating blood from fat, ruling out fat-containing lesions such as teratomas, and highly sensitive for the detection of haemorrhagic foci in endometriosis. DWI sequences are helpful in the characterisation of fluids: hypercellular fluids, such as pus, show restricted diffusion and therefore appear hyperintense on DWI images and hypointense on the corresponding apparent diffusion coefficient (ADC) map. According to the European Society of Urogenital Radiology (ESUR) guidelines, gadolinium-enhanced fat-suppressed T1-weighted sequences are useful (a) to assess a lesion’s contrast enhancement pattern, (b) to depict enhancing mural nodules within adnexal masses, (c) to identify contrast extravasation indicating active bleeding, (d) to differentiate PID from endometriosis, (e) to identify inflammatory enhancement of perivisceral fat and peritoneum and (f) for characterisation of leiomyomas and differential diagnosis from leiomyosarcomas and adnexal masses [13, 16]. Finally, delayed urographic acquisitions allow preoperative depiction of the ureters and their anatomical relationship to adnexal masses [17].

**Table 1:** State-of-the-art MRI protocol for comprehensive study of the female pelvis. Synoptic table summarizes the pulse sequence parameters. If not contraindicated, preliminary intramuscular or intravenous administration of an antispasmodic agent (e.g. N-butyl-scopolamine) may be performed. Axial T2-weighted SSFSE sequence is used as a second localiser. Sagittal T2-weighted FRFSE sequence is planned parallel to the long axis of the uterus, identified on the axial T2-weighted SSFSE sequence. Oblique-coronal and oblique-axial T2-weighted FRFSE sequences are planned parallel and perpendicular to the long axis of the uterus, respectively. Sagittal, oblique-coronal and oblique-axial T1-weighted 3D gradient-echo liver acquisition with volume acceleration (LAVA) sequences with fat suppression are obtained before and after intravenous administration of gadolinium-based contrast agent (0.1 mmol/kg, followed by 20 ml of saline solution, both at a 2 ml/s flow rate); in particular, the sagittal sequence is acquired at 60 and 120 s after contrast media administration. If needed, an additional urographic phase T1-weighted 3D gradient-echo LAVA sequence may be obtained in the sagittal, coronal and axial planes approximately 10 min after contrast administration. T2-weighted FRFSE, T2* and DW sequences are acquired with free-breathing technique, whereas T1-weighted 3D gradient-echo LAVA sequences are acquired in breath hold. T2* represents an optional sequence, not routinely performed

MRI protocol	Axial T2W SSFSE	Sagittal T2W FRFSE	Oblique-coronal/axial T2W FRFSE	Axial T1W GRE in-out (chemical shift)	Axial T2*	Axial DWI SE EPI	Sagittal, oblique-coronal/axial T1W 3D GRE LAVA
Repetition time/echo time (ms)	765/59	4675/100	4675/100	180/2.1	650/11.8	3000/74.1	4.4/2.1
Flip angle	90°	90°	90°	80°	20°	90°	12°
Section thickness (mm)	6	4	4	6	5	5	3.4
Interslice gap (mm)	0.6	0.4	0.4	0.6	0	1	−1.7
Bandwidth (kHz)	31.25	41.67	41.67	62.5	8.33	250	62.5
Field of view (cm)	38	32	32	38	25	46	40
Matrix	320 × 288	320 × 224	320 × 224	256 × 224	256 × 256	160 × 160	320 × 192
No. of averages	0.54	4	4	1	1	16	0.75
No. of images	30	26	26	20	30	28	104
Frequency direction	Right to left	Anterior to posterior	Right to left		Anterior to posterior	Anterior to posterior	Superior to inferior
Acquisition time	24 s	3 min 49 s	3 min 49 s	22 s	1 min 28 s	3 min 18 s	22 s
*b* value (s/mm^2^)	–	–	–	–	–	0–800	–

*T2W* T2-weighted, *T1W* T1-weighted, *SSFSE* single-shot fast spin-echo, *FRFSE* fast recovery fast spin-echo, *DWI* diffusion-weighted imaging, *SE* spin-echo, *EPI* echoplanar imaging, *GRE* gradient-echo, *LAVA* liver acquisition with volume acceleration

Although very complete, the abovementioned protocol (Table 1) lasts approximately 30 min (40 min including urographic acquisitions) and seems hardly applicable in an emergency setting. When dealing with ill or non-cooperating patients with acute pelvic pain, we suggest adopting a time-efficient non-contrast-enhanced MR imaging protocol that relies on faster sequences, which lasts approximately 15 min (Table 2).

**Table 2:** Time-efficient noncontrast MRI protocol for urgent study of the female pelvis and non-cooperating patients

MRI protocol	Axial T2W SSFSE	Sagittal T2W FRFSE	Oblique coronal/axial T2W FRFSE	Axial DWI SE EPI	Sagittal, oblique coronal/axial T1W 3D GRE LAVA
Repetition time/echo time (ms)	765/59	4675/100	4675/100	3000/74	4.4/2.1
Flip angle	90°	90°	90°	90°	12°
Section thickness (mm)	6	4	4	8	3.4
Interslice gap (mm)	0.6	0.4	0.4	2	−1.7
Bandwidth (kHz)	31.25	41.67	41.67		62.5
Field of view (cm)	38	32	32	42	40
Matrix	320 × 288	320 × 224	320 × 224	160 × 160	320 × 192
No. of averages	0.54	2	2	2	0.75
No. of images	30	26	26	15	104
Frequency direction	Right to left	Anterior to posterior	Right to left	Anterior to posterior	Superior to inferior
Acquisition time	24 s	2 min 15 s	2 min 15 s	27 s	22 s
*b* value (s/mm^2^)	–	–	–	0–800	–

*T2W* T2-weighted, *T1W* T1-weighted, *SSFSE* single-shot fast spin-echo, *FRFSE* fast recovery fast spin-echo, *DWI* diffusion-weighted imaging, *SE* spin-echo, *EPI* echoplanar imaging, *GRE* gradient-echo, *LAVA* liver acquisition with volume acceleration

On MRI pelvic examinations, orthopaedic metal implants such as hip prosthesis may determine signal loss, geometric distortion, and failure of fat suppression. Metal artefact reduction sequences (MARS) is a general definition which does not refer to a single specific technique, but rather encompasses a variety of sequences optimised to reduce artefacts from metal. As a general consideration, artefacts are less severe at 1.5 T than at 3 T, since the susceptibility-induced field inhomogeneity is doubled at 3 T MR scanners. As for conventional techniques, turbo spin echo are preferable to gradient echo sequences, and short tau inversion recovery (STIR) performs better than standard spectral fat saturation.

Other tricks to optimise standard sequences, although often at the expense of acquisition time, include increasing receiver bandwidth, increasing matrix size, decreasing echo times and decreasing slice thickness. In recent years, new dedicated metal artefact reduction techniques have been developed including view angle tilting, the multispectral imaging techniques, multiacquisition variable-resonance image combination and slice-encoding for metal artefact correction. In patients with metal implants, these sequences, associated with advanced image acquisition techniques such as parallel imaging, allow to acquire MARS in a clinically feasible scan time [18].

### MRI interpretation

In reproductive age females, the normal ovary shows homogenous soft-tissue T1-weighted signal intensity and is often barely perceptible from adjacent bowel. On the other hand, on T2-weighted images, ovaries are almost always identifiable on the basis of characteristic zonal anatomy including a low signal intensity cortex and high signal intensity medulla with scattered well-circumscribed fluid foci corresponding to follicles. The normal, non-dilated fallopian tubes are usually hardly recognizable at MRI unless outlined by pelvic fluid. In the presence of peritoneal effusion, the fallopian tubes become visible as paired, thin and serpentine juxtauterine structures. When dilated by serous fluid, haemorrhage or pus, fallopian tubes appear as fluid-filled tubular structures that arise from the upper lateral margin of the uterine fundus, showing incomplete parietal plicae or folds that give them the characteristic convoluted appearance [19].

## Haemorrhagic ovarian cysts

### Physiology

In reproductive age females, the majority of ovarian cysts are physiological or functional in nature and encompass dominant follicles, follicular cysts and luteal cysts. During the follicular phase of menstrual cycle, under oestrogen stimulation, ovarian follicles grow until only one in each cycle reaches a size of approximately 20–25 mm, becoming the dominant follicle. Sometimes a dominant follicle fails to ovulate, but remains hormonally sensitive and continues to grow becoming a follicular cyst. During ovulation, the dominant follicle ruptures, releases the oocyte and then collapses; at this point, the luteinising hormone causes proliferation of granulosa cells in the inner lining of the remaining follicular bed, resulting in the formation of the corpus luteum.

**Fig. 1:**
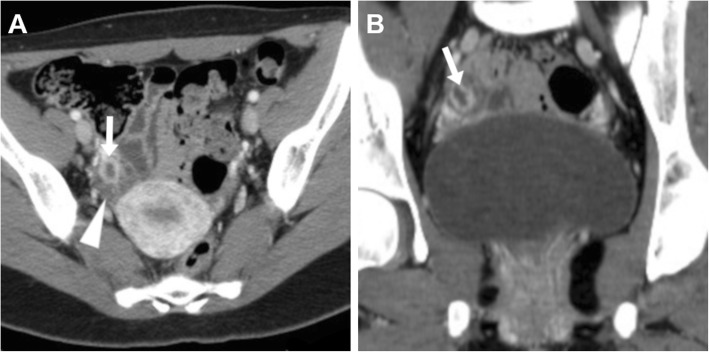
Typical appearance of an uncomplicated corpus luteum in the second half (luteal phase) of the menstrual cycle on axial (**a**) and coronal (**b**) CT images: the normal-sized right ovary (arrowhead) contains a 1.5-cm cystic structure demarcated by a crenulated, strongly enhancing peripheral rim (arrow)

**Fig. 2:**
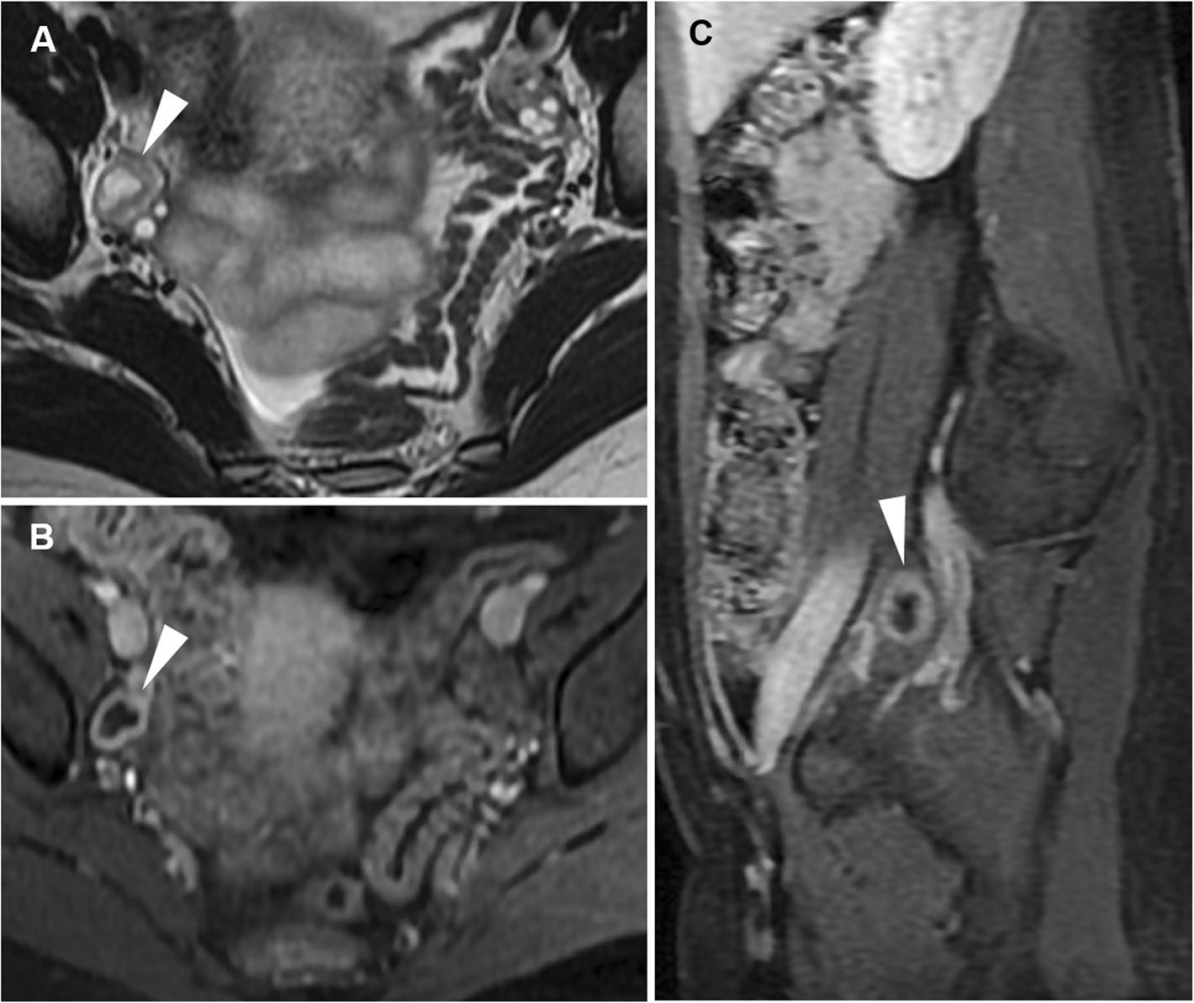
Usual MRI appearance of an uncomplicated corpus luteum in a 26-year-old woman. Oblique-coronal T2-weighted (**a**), oblique-coronal (**b**) and sagittal (**c**) gadolinium-enhanced fat-suppressed T1-weighted images show a small-sized, unilocular fluid-containing structure in the right ovary (arrowheads), with typical homogenous T1-hypointense signal and T2-hyperintense signal, thickened walls with a crenulated enhancing rim

A functional structure which regresses spontaneously when pregnancy does not occur, the corpus luteum is frequently seen when cross-sectional imaging is performed in the second part (luteal phase) of the menstrual cycle. The CT hallmark of a corpus luteum is a well-circumscribed, unilocular (rarely bilocular) cystic structure located within the ovary, which measures up to 3 cm and is demarcated by a crenulated, briskly enhancing rim that reflects prominent blood flow within thickened walls (Fig. 1) and corresponds to the colour Doppler US ‘ring of fire’ sign. MRI shows a similar appearance with internal fluid-like hypointense and hyperintense signal on T1- and T2-weighted images, respectively, and intense wall enhancement after gadolinium administration (Fig. 2) [8, 9, 20].

**Fig. 3:**
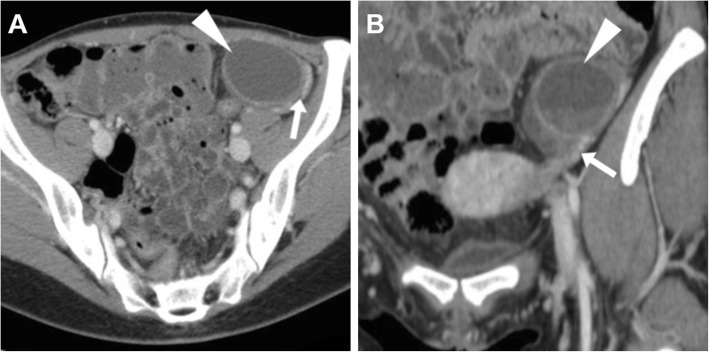
Uncomplicated, symptomatic follicular ovarian cyst causing left lower quadrant pain. Axial (**a**) and coronal (**b**) CT images show a 4 × 3 cm cystic mass (arrowheads) with homogeneous fluid attenuation, laterally displaced in the left hemipelvis. Identification of the ovarian vessels in the suspensory ligament (arrows) helps in confirming the adnexal origin of the cyst

**Fig. 4:**
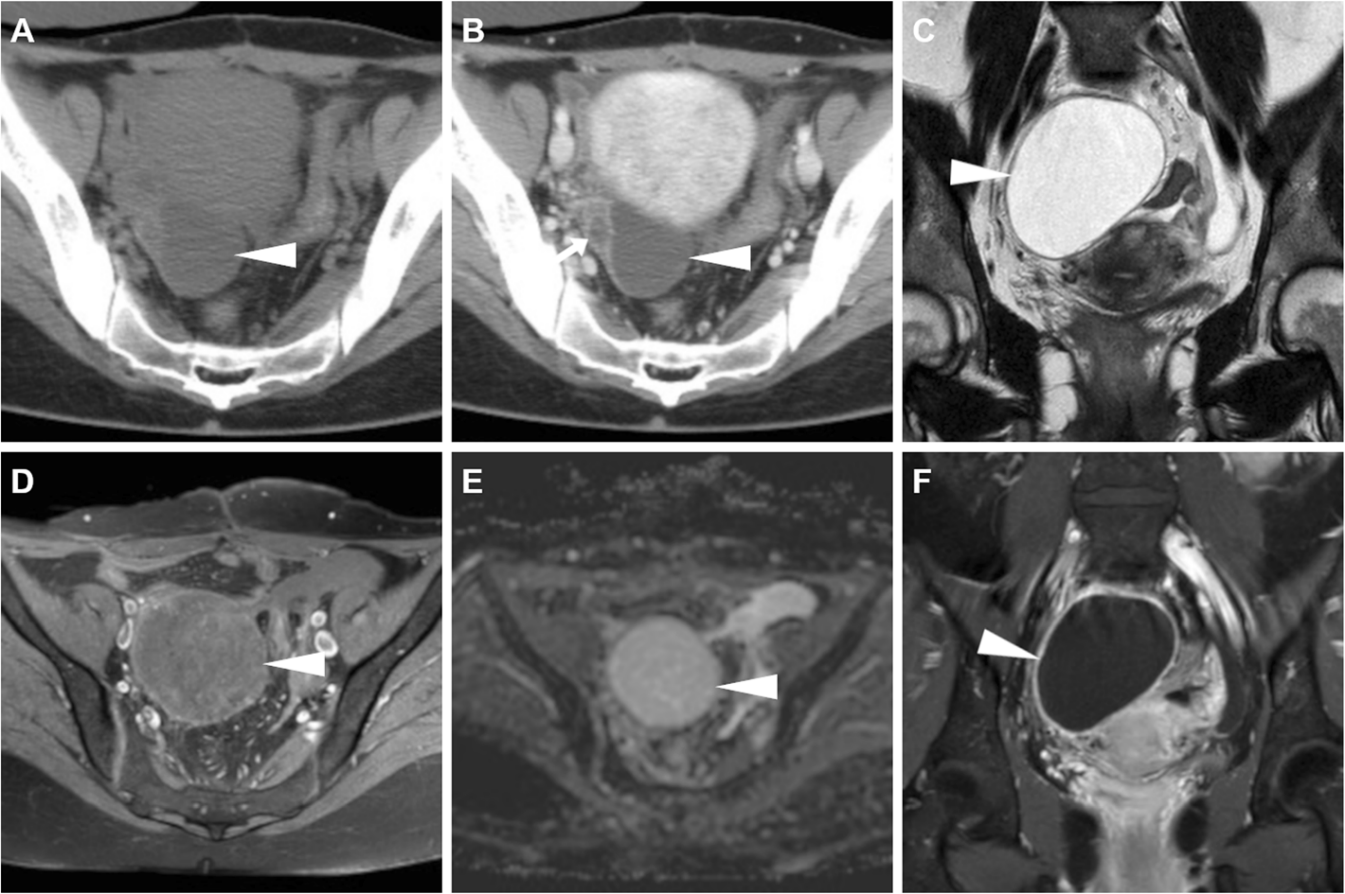
A surgically proven, progressively enlarging ovarian cyst. Initial unenhanced (**a**) and post-contrast (**b**) CT images depicted a 4 × 3 cm non-enhancing retrouterine cystic lesion (arrowheads) demarcated by a thin regular wall. Weeks later, MRI showed cyst enlargement with homogeneous T2-weighted fluid signal (**c**), without evidence of blood on precontrast fat-suppressed T1-weighted sequence (**d**), with unrestricted diffusion on apparent diffusion coefficient map (**e**) and thin uniform enhancing wall on post-gadolinium acquisition (**f**)

**Fig. 5:**
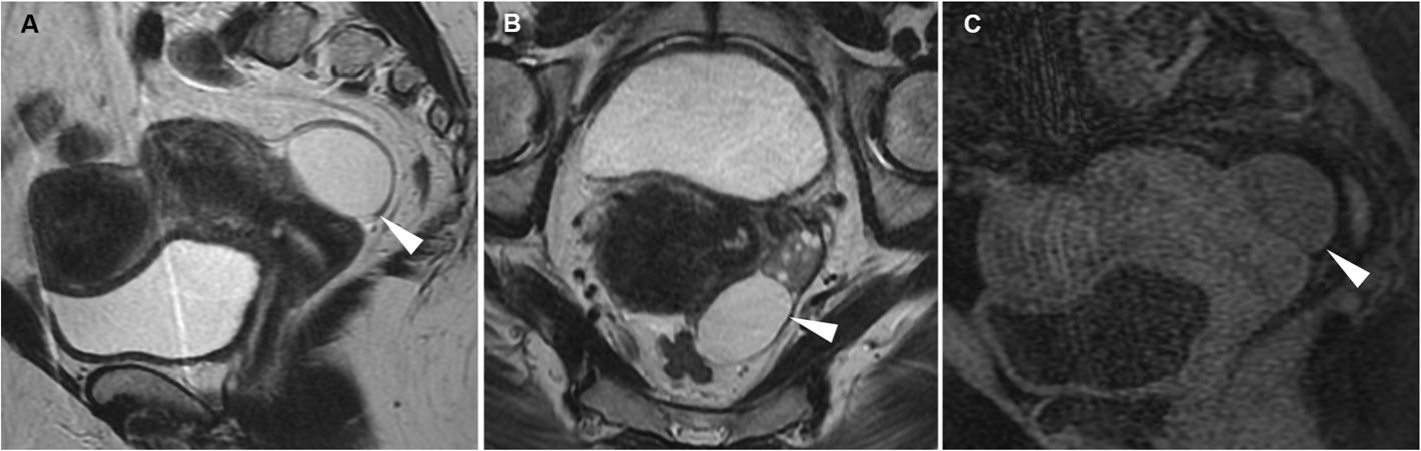
MRI of a functional cyst causing pelvic pain in the periovulatory phase in a 37-year-old woman. Sagittal (**a**) and oblique-axial (**b**) T2-weighted images show a 4-cm well-marginated, unilocular cystic lesion with thin walls and homogeneous fluid-like signal intensity arising from the posterior edge of the left ovary (arrowheads), without haemorrhagic hyperintensity on sagittal precontrast fat-suppressed T1-weighted image (**c**)

When a corpus luteum fails to regress, a luteal cyst develops and may be filled with either fluid or blood, so the terms corpus luteum cyst and haemorrhagic corpus luteum are often used as synonyms [21]. At CT, both follicular and corpus luteum cysts present as well-marginated unilocular lesions, usually measuring less than 5 cm, with thin walls and homogeneous fluid-like attenuation (Figs. 3 and 4). A 3-cm threshold has been suggested to differentiate follicles and corpus luteum (below 3 cm) from functional (follicular/luteal) cysts (over 3 cm). At MRI, ovarian cysts containing simple fluid have prolonged T1 and T2 relaxation times and therefore show homogeneously low T1-weighted and very high T2-weighted signal intensity (Figs. 4 and 5) [22].

### Haemorrhagic ovarian cysts

**Fig. 6:**
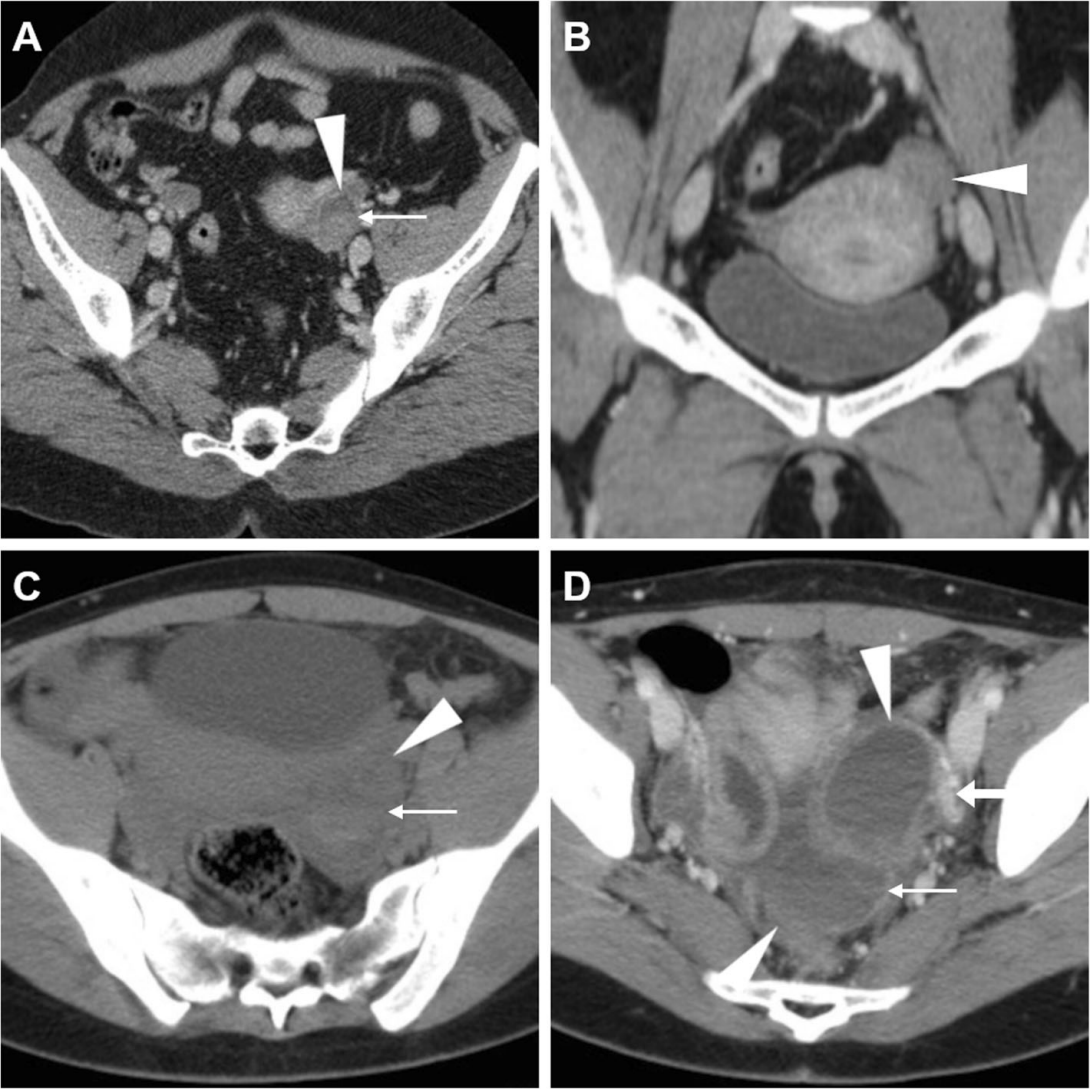
Two CT cases of haemorrhagic ovarian cysts. **a**, **b** The normal-sized left ovary (arrowheads) show mixed appearance with dependent high attenuation and fluid-fluid level (thin arrow in **a**) consistent with haemorrhagic corpus luteum. **c**, **d** Unenhanced (**c**) and post-contrast (**d**) images show a larger, bilocular adnexal lesion (arrowheads) with thin regular peripheral enhancement and dependent hyperattenuation (thin arrows). Note the ovarian vessels (arrow in **d**)

**Fig. 7:**
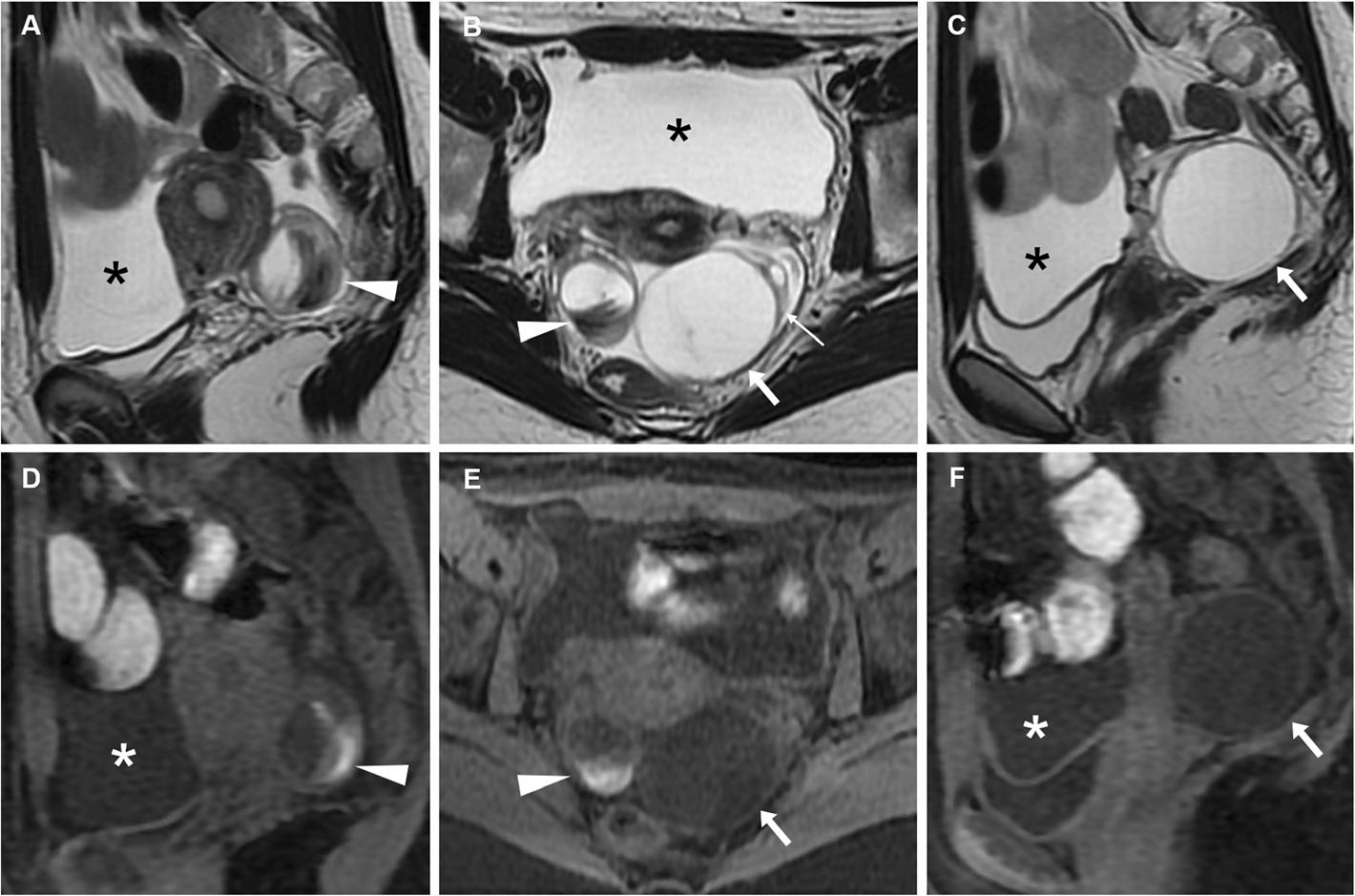
Haemorrhagic corpus luteum and functional cyst in a 15-year-old woman with pelvic pain. Multiplanar T2-weighted (**a**–**c**) and precontrast fat-suppressed T1-weighted (**d**–**f**) images showed a right adnexal cyst (arrowheads) with internal fluid-fluid level and a bloody dependent component, T2-hypointense and T1-hyperintense. Contralaterally, a well-marginated unilocular cystic lesion of the left ovary (arrows) shows thin walls and homogeneous fluid-like signal intensity. Note the compressed ovarian parenchyma along the lateral edge of the cyst (thin arrow in **b**) and pelvic peritoneal effusion (asterisk)

Unilateral acute pelvic pain and tenderness leading to ED admission may result from cyst enlargement, haemorrhage or rupture. Bleeding within luteal cyst or other functional cyst results in the formation of a haemorrhagic ovarian cyst. In the former case, bleeding is caused by the hypervascularity and fragile vessels of the granulosa layer within the wall of the cyst. At CT, the presence of blood leads to the appearance of increased attenuation (> 40 Hounsfield units, HU) within the cyst. Haemorrhagic ovarian cysts are unilateral, show mixed (in the range of 25–100 HU) internal attenuation with common fluid-debris or fluid-fluid levels (Fig. 6) and often appear as complex masses. Considerable overlap exists between imaging appearances of haemorrhagic follicular and luteal cysts, and differentiation among the two entities should rely on the current phase of the menstrual cycle. After intravenous contrast, the walls of luteal cysts appear thicker than those of follicular cysts and highly vascularised. The MRI pattern of haemorrhagic cysts depends on the age of the blood, the amount of serous fluid and clot formation and retraction (Fig. 7). The most usual appearance is that of a unilocular, unilateral cyst with relatively high signal intensity on T1-weighted images and variable, heterogeneous T2-weighted signal intensity; intracystic fluid-fluid levels are commonly seen [8, 23].

### Differential diagnosis

**Fig. 8:**
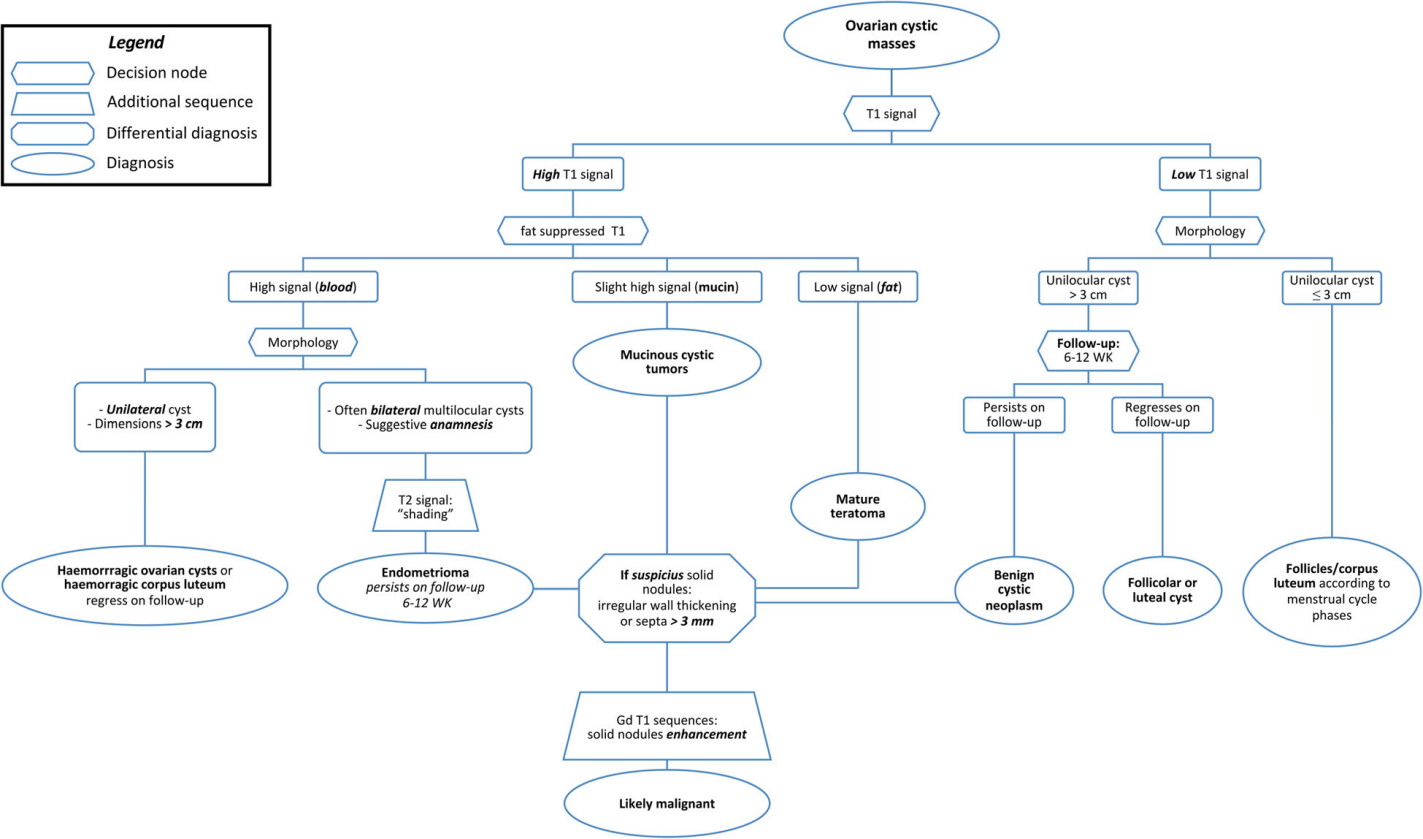
Flow-chart showing the MRI algorithm summarising the diagnostic steps for elucidating cystic ovarian masses

Table 3 summarises the CT and MR imaging features of ovarian cysts. Additionally, a flowchart summarising the steps of an algorithm for MRI differential diagnosis of ovarian cystic masses is proposed in Fig. 8.

**Table 3:**
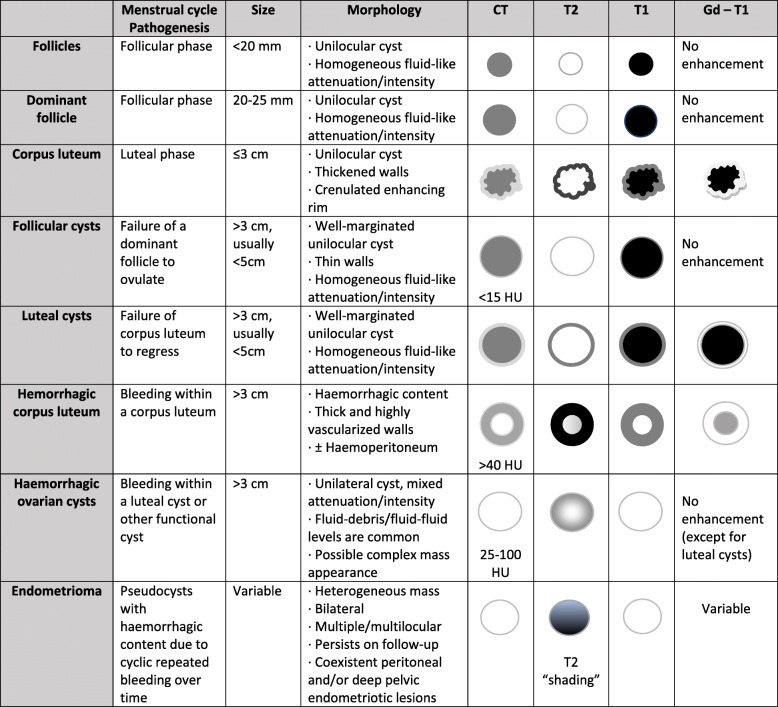
Chart summarising CT and MR imaging findings of ovarian cysts

The endometrioma represents the key differential diagnosis of haemorrhagic ovarian cysts. Affecting 10% of women of reproductive age, endometriosis is defined by growth of ectopic endometrial tissue outside the uterus, which bleeds synchronously with the endometrium. Clinically, endometriosis is suggested by cyclic perimenstrual pain and by other manifestations such as dysmenorrhoea, dyspareunia, infertility and pain during defecation. Although dysmenorrhoea and chronic pelvic pain represent the classic clinical presentation, a sudden onset with acute pelvic pain may also occur [24]. Because of the great clinical variability of the disease, delayed diagnosis up to 7–10 years after initial symptoms is common [25].

**Fig. 9:**
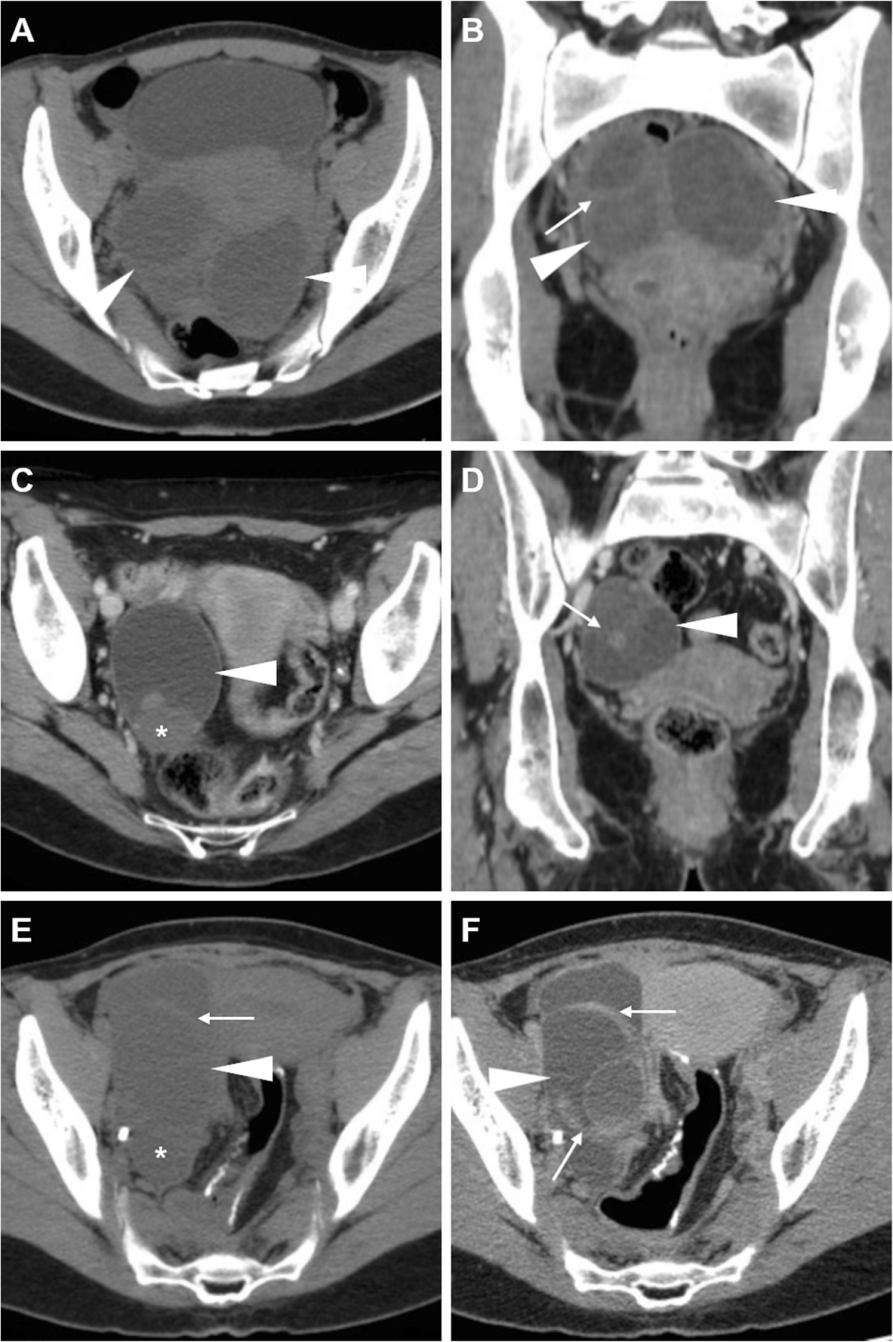
Key CT differential diagnoses of haemorrhagic ovarian cysts. **a**, **b** Unenhanced (**a**) and postcontrast (**b**) CT images of bilateral endometriomas (arrowheads) occupying the rectouterine pouch in a ‘kissing ovaries’ configuration. Note the mildly inhomogeneous attenuation and septation (thin arrow in **b**). **c**, **d** Ovarian carcinoma in a premenopausal woman with predominantly cystic pattern, septations (thin arrow in **d**) and a solid, enhancing eccentric mural vegetation (asterisk in **c**). **e, f** Unenhanced (**e**) and post-contrast (**f**) images of a postoperative collection (complex serocele, arrowheads) involving the adnexal region, showing septations (thin arrows). Note the right ureteral stent and suture at the sigmoid colon after multiple surgeries for Crohn’s disease

More common (80% of patients) compared to superficial peritoneal implants and deep pelvic endometriosis, endometrioma results from ectopic endometrial glands in the ovarian cortex that form haemorrhagic ‘pseudocysts’ which become detectable by imaging. Endometriomas have highly variable, often heterogeneous CT appearance and often mimic mixed solid/cystic or predominantly solid masses (Fig. 9a, b). Compared to MRI, CT has poor specificity and often does not allow reliable differentiation from other complex cystic adnexal masses, either benign or malignant (Fig. 9). Suggestive ancillary findings include multiplicity, bilateral adnexal involvement (‘kissing ovaries’ sign, Fig. 9a, b), stability over time and associated lesions on the peritoneum and bowel serosa [8, 17].

**Fig. 10:**
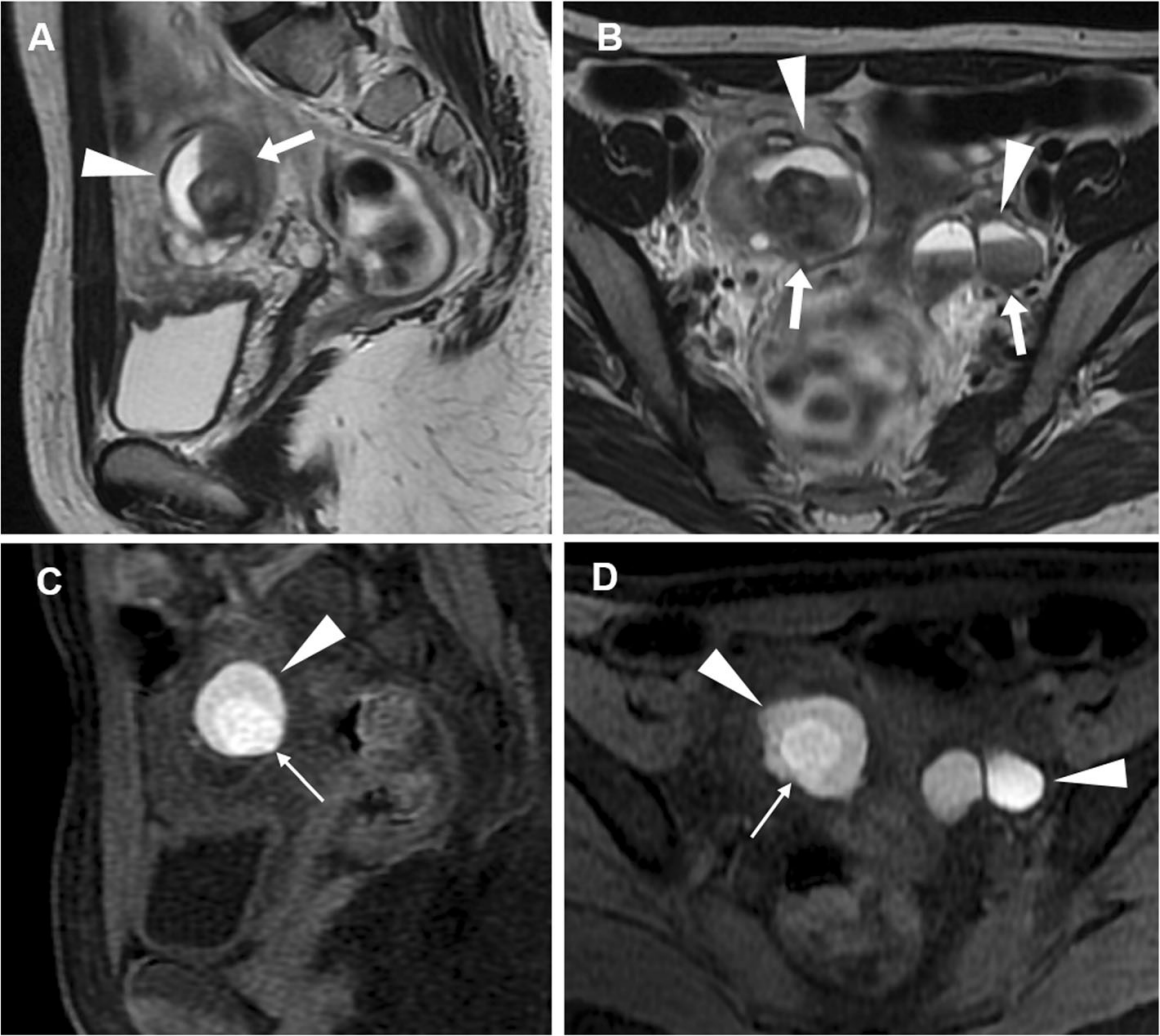
MRI of bilateral endometriomas in a 27-year-old woman with pelvic pain exacerbated in the last few days. Sagittal (**a**) and oblique-coronal (**b**) T2-weighted images show bilateral endometriomas with fluid-blood levels (arrowheads) and low signal intensity in the declivous portion of the cyst (‘shading sign’, arrows). On sagittal (**c**) and oblique-coronal (**d**) precontrast fat-suppressed T1-weighted images, the cysts demonstrate high signal intensity (arrowheads). Note the clot within right endometrioma with very high T1 signal intensity (thin arrows)

**Fig. 11:**
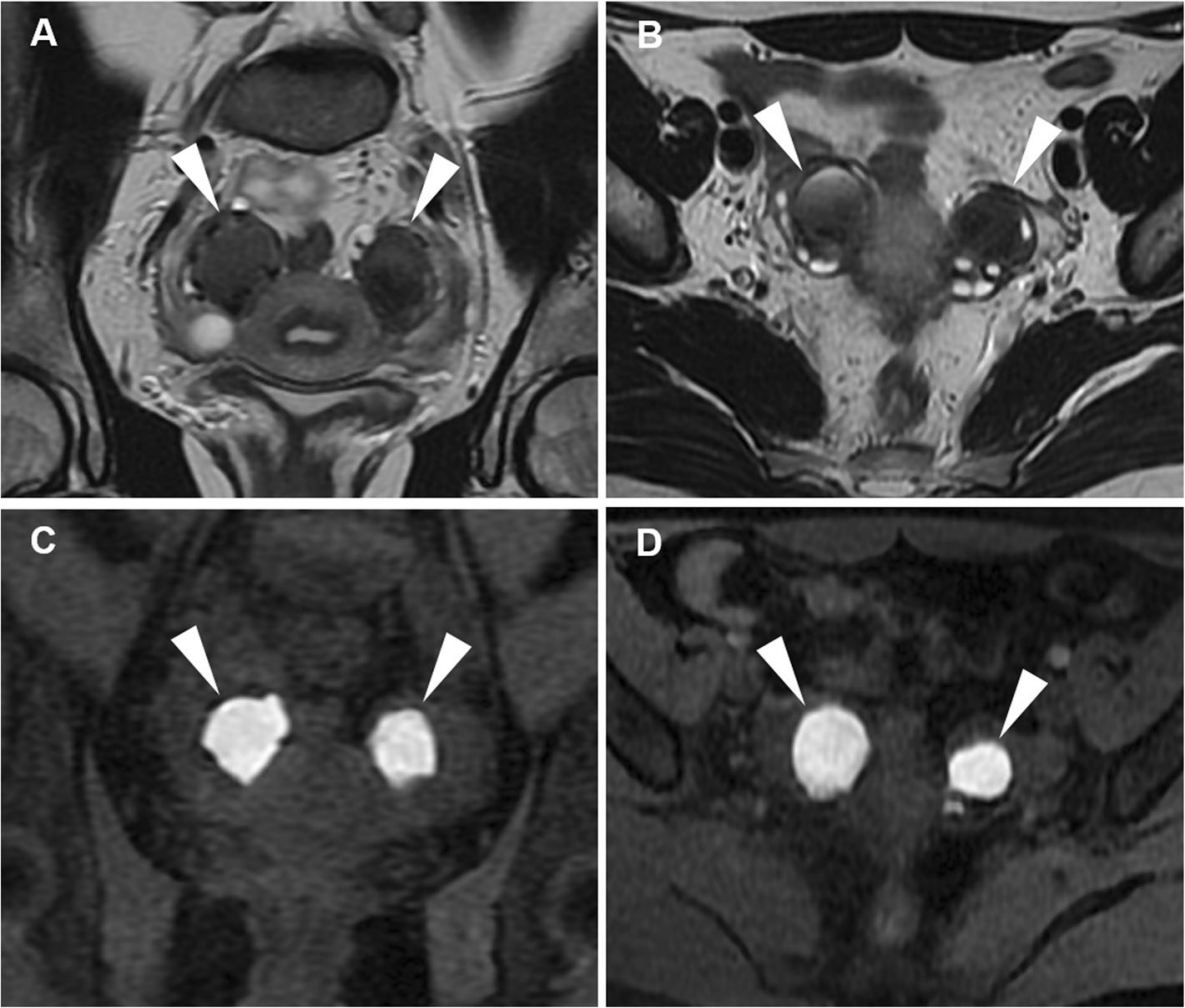
MRI of bilateral endometriomas in a 31-year-old woman suffering from lumbar and pelvic pain. Oblique-axial (**a**) and oblique-coronal (**b**) T2-weighted images show bilateral endometriomas (arrowheads) with variable grade of low signal intensity until complete signal loss (‘shading sign’). On oblique-axial (**c**) and oblique-coronal (**d**) precontrast fat-suppressed T1-weighted images, the cysts display homogeneous high signal intensity (arrowheads)

MRI is arguably the best imaging technique for the diagnosis of endometrioma, relying on high signal intensity on T1-weighted images and the pathognomonic T2 ‘shading’ sign (gradual loss of signal till complete signal void in the most dependent portion) reflecting cyclic repeated bleeding over time (Figs. 10 and 11). Haemorrhagic ovarian cyst is suggested over endometrioma when the cyst is solitary and unilocular, without the ‘shading sign’, and disappears at follow-up (6–12 weeks). Other T1-hyperintense lesions which may be confused with endometriomas include dermoids and mucinous cystic tumours: precontrast fat-suppressed T1-weighted images help distinguish lipid-containing dermoids from the blood. Mucinous cystic tumours show signal intensity lower than the blood on T1-weighted images. Finally, the identification of vascularised projections and nodular septa should suggest malignancy [17, 22, 26, 27].

In this regard, it is important to remember the importance of post-processing images when facing a cystic mass that is hyperintense on unenhanced T1-weighted fat-suppressed sequences. Subtraction techniques that suppress the T1 hyperintense background of the cystic content allow to detect true gadolinium enhancement in suspicious solid mural nodules (a finding suggesting tumour arising from endometrioma), which may be hardly identifiable at visual assessment alone [14, 28].

## Gynaecologic causes of haemoperitoneum

### Bleeding corpus luteum

In women of reproductive age, the genital tract is the most common source of non-traumatic haemoperitoneum and bleeding corpus luteum represents the leading cause, secondary to the high vascularity of the luteal walls [29, 30]. This potentially life-threatening condition is rare in adolescence and occurs at a median age 26 years, often (nearly 60% of cases) after sexual intercourse or physical exercise. The right ovary is more frequently involved because the contralateral one is protected by the sigmoid colon ‘cushion’. The usual presentation includes a sudden onset of pelvic pain which gradually spreads to the entire abdomen, ultimately leading to peritoneal irritation and even life-threatening shock. Amenorrhoea, vaginal bleeding, early pregnancy signs and elevated beta human chorionic gonadotropin (β-hCG) are absent [31, 32].

**Fig. 12:**
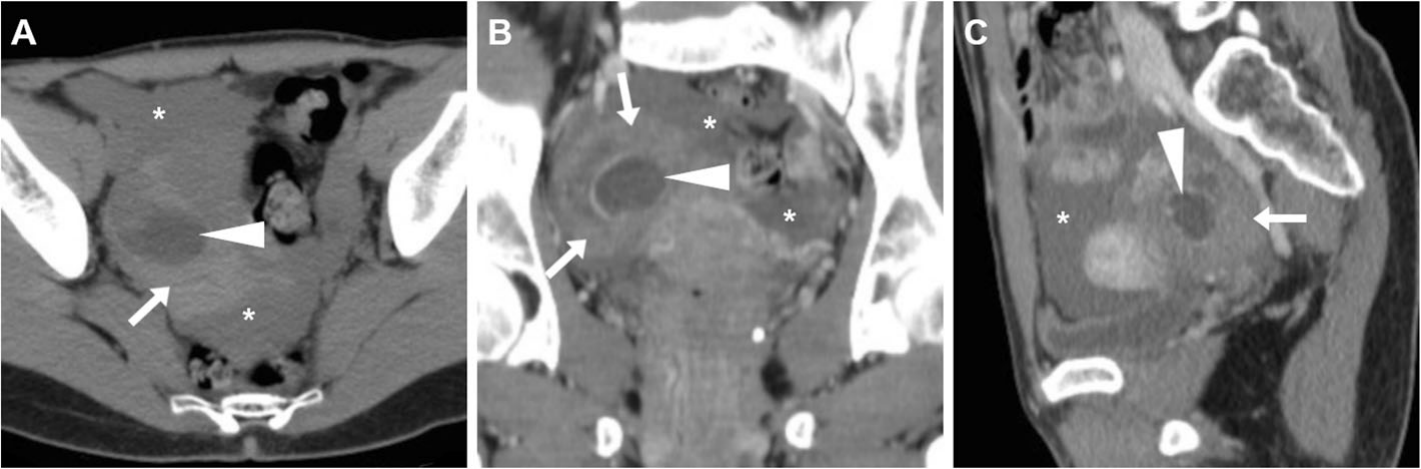
Haemoperitoneum shown at CT (**a**–**c**) as pelvic effusion (asterisk) with attenuation higher than that of water, caused by ruptured corpus luteum seen as a 3-cm right adnexal cystic lesion (arrowheads) with enhancing wall, surrounded by high-attenuation ‘sentinel clot’ (arrows)

**Fig. 13:**
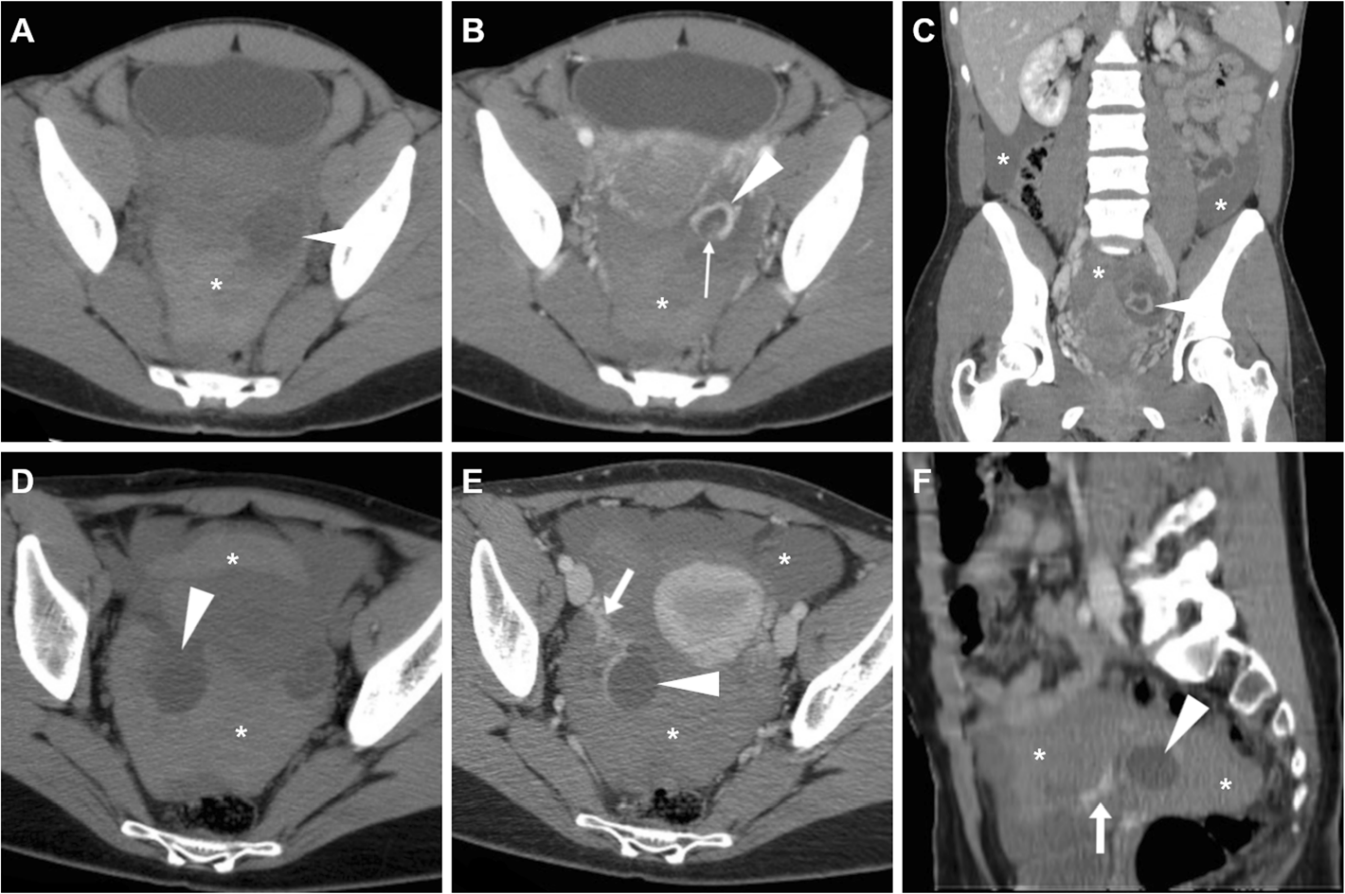
Two cases of haemoperitoneum from bleeding corpus luteum. **a**–**c** Unenhanced (**a**) and post-contrast (**b**, **c**) CT show hyperattenuating effusion (asterisk) in pelvis and paracolic gutters, mildly enlarged left ovary containing a characteristic corpus luteum (arrowheads) with focal discontinuity (thin arrow in **b**) of its strongly enhancing crenulated wall. **d**–**f** Unenhanced (**d**) and post-contrast (**e**, **f**) CT images show pelvic blood (asterisk) surrounding a 3-cm cystic lesion of the right ovary (arrowheads), from which contrast extravasated (arrows in **e** and **f**) indicating active bleeding

At CT, the ruptured corpus luteum is seen as a shrunken ovarian cyst with discontinuity of the thickened wall, often surrounded by hyperattenuating ‘sentinel clot’. Occasionally, active bleeding may be detected as serpiginous or jet-like contrast extravasation adjacent to the cyst, most usually in the portal phase reflecting intermittent or venous haemorrhage (Figs. 12 and 13). Haemoperitoneum appears as hyperdense (> 30 HU) peritoneal-free fluid, with higher attenuation typically detected within the dependent portion of peritoneal cul-de-sac (Figs. 12 and 13) [3, 8, 20, 29, 30].

**Fig. 14:**
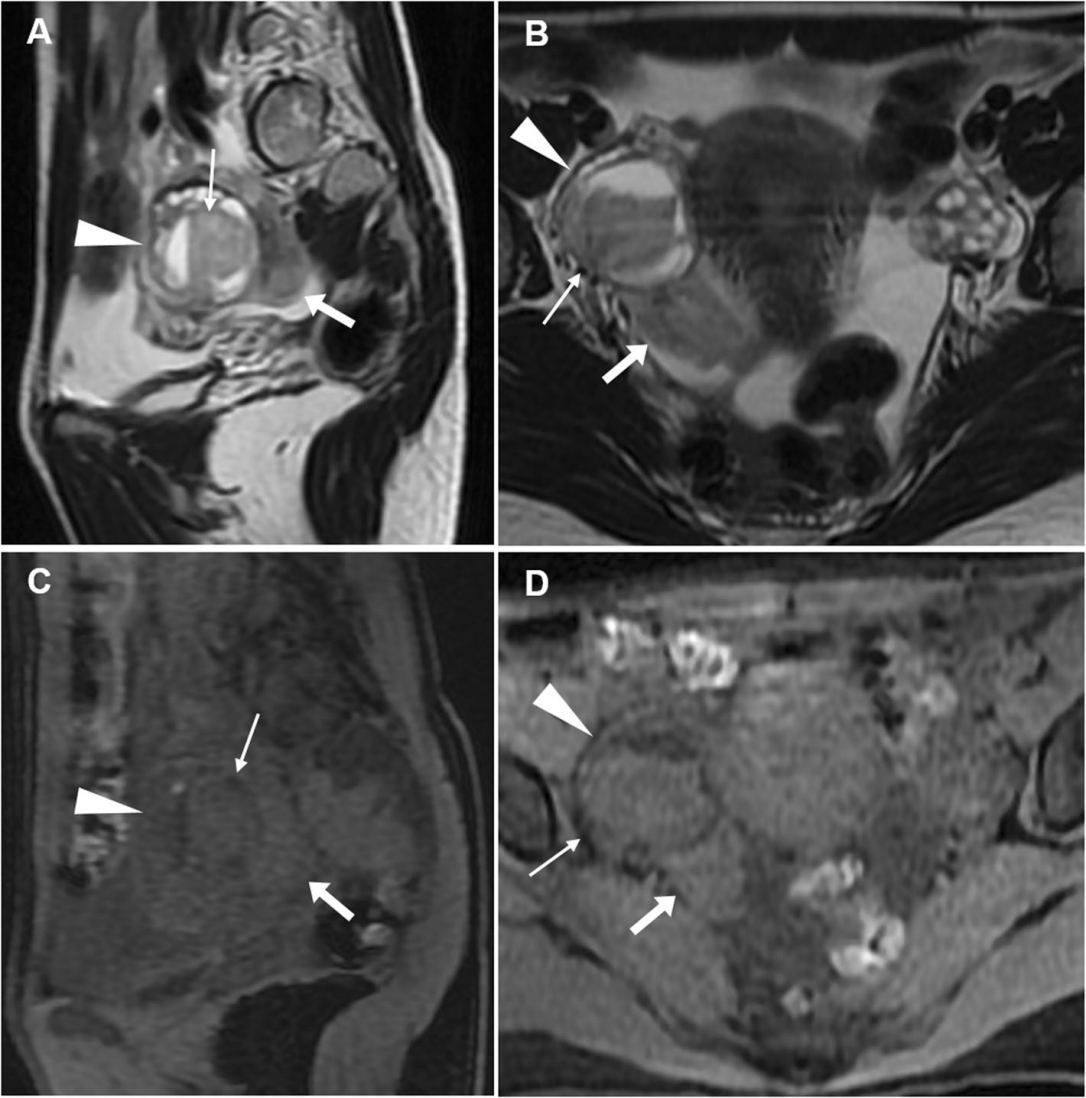
Ruptured haemorrhagic corpus luteum with haemoperitoneum in an 18-year-old woman with pelvic pain for 24 h. Sagittal (**a**) and oblique-coronal (**b**) T2-weighted, sagittal (**c**) and oblique-coronal (**d**) precontrast fat-suppressed T1-weighted images show right-sided ovarian cyst (arrowheads) with internal fluid-fluid level and a dependent component with intermediate signal intensity on T1- and T2-weighted images (thin arrows), corresponding to recent haemorrhage (< 48 h). Note the haemorrhagic peritoneal effusion with intermediate signal intensity on both T1 and T2-weighted images adjacent to the posterior aspect of the cyst (arrows)

**Fig. 15:**
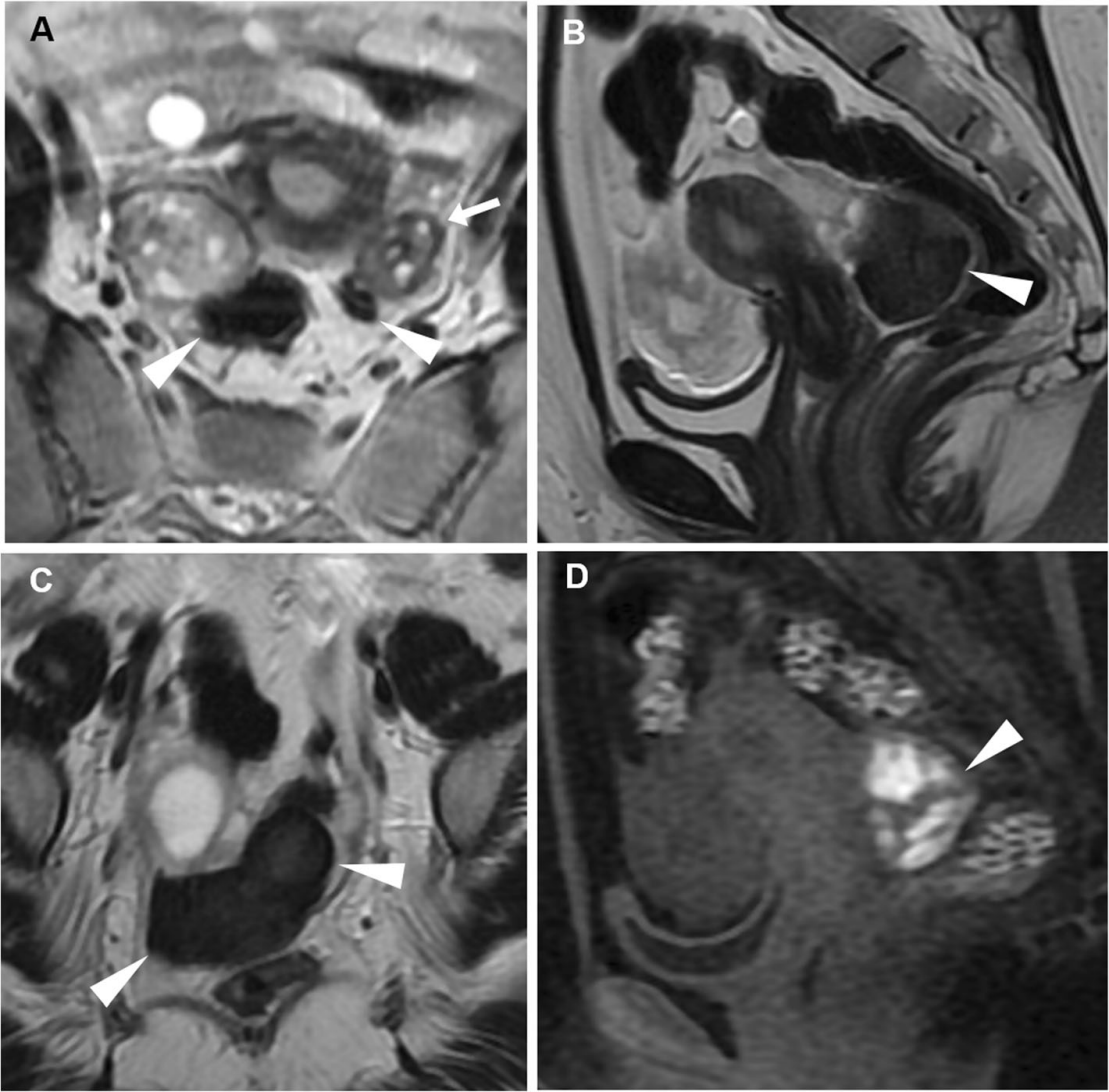
Ruptured corpus luteum in a 20-year-old woman with pelvic pain and fever over the previous 2 days. Oblique-axial (**a**), sagittal (**b**) and oblique-coronal (**c**) T2-weighted and sagittal precontrast fat-suppressed T1-weighted (**d**) images show a haemorrhagic collection (arrowheads), T2-hypointense and T1-hyperintense, in the pouch of Douglas, contiguous to the left ovary (arrow in **a**). The latter represents the source of bleeding, although the corpus luteum is no longer recognizable

At MRI, haemoperitoneum shows variable signal intensity depending on its age (haemoglobin degradation stages), the extent of the bleeding and blood clot formation. Within 48 h from bleeding, pelvic intraperitoneal haemorrhage generally shows intermediate signal intensity (higher than that of urine) on T1-weighted images and intermediate T2-weighted signal intensity (Fig. 14). Later, a fluid-fluid level can be seen, with the dependent portion of the bloody ascites being hyperintense compared with the supernatant on T1-weighted images; on T2-weighted images, the signal intensity relationship is reversed. This appearance corresponds to the CT ‘haematocrit effect’, where sedimented erythrocytes produce a dependent layer of high attenuation. Intraperitoneal haemorrhage may contain localised clot showing higher signal intensity than the free intraperitoneal blood on T1-weighted images and low signal intensity on T2-weighted images. These clots may be useful in identifying the bleeding source since they generally form close to the organ from which the haemorrhage originated (‘sentinel clot’ sign) (Fig. 15). Compared to CT, active haemorrhage is occasionally identified on MRI as hyperintense blush on fat-suppressed T1-weighted images after gadolinium administration [8, 9].

Whereas in the past, the majority of cases were treated surgically, nowadays, in haemodynamically stable patients, the management approach is increasingly conservative with close observation and roughly 20% of patients ultimately require laparoscopic surgery [31, 32]. Some authors proposed the use of CT to predict the clinical outcome, stating that in their experience, active bleeding and massive pelvic haemoperitoneum (anteroposterior diameter > 6 cm) dictate the need for operative treatment [20, 33].

### Ectopic pregnancy

The second most frequent gynaecologic cause of haemoperitoneum, ruptured ectopic pregnancy (EP) accounts for 6% of pregnancy-related mortality. Mostly encountered in the fourth decade of life, EP occurs in approximately 1.3–2.4% of all pregnancies and refers to the implantation of a blastocyst at a different location from the endometrium of uterine cavity, most usually in the fallopian tube (about 95% of all ectopic pregnancies, involving the ampulla, isthmus and fimbria in descending order of incidence). The other uncommon implantation sites are listed in Table 4 [34, 35]. Predisposing factors include prior EP, history of tubal surgery, scarring related to previous PID and infertility treatments such as in vitro fertilisation and embryo transfer. The presentation includes acute abdominal pain closely similar to that produced by ruptured corpus luteum, but associated with amenorrhoea, vaginal bleeding and positive beta human chorionic gonadotropin (β-hCG) [34].

**Table 4:** Rare non-tubal implantation sites of ectopic pregnancy (EP)

Site	Frequency	Features
Interstitial EP	2–4% of cases	Implantation of the gestational sac in the interstitial or intramyometrial segment of the fallopian tube
Ovarian EP	Up to 3% of cases	Fertilized ovum is retained in the ovary
Abdominal EP	0.9–1.4% of cases	Implantation of fertilised ovum anywhere on the peritoneal surface or abdominal viscera
Cornual EP	< 1% of cases	Implantation of a blastocyst within the cornua of a bicornuate or septate uterus
Cervical EP	< 1% of cases	Implantation of the blastocyst within the endocervical canal
Caesarean scar EP	< 1% of cases	Implantation of a blastocyst at the site of a prior caesarean scar
Intramural EP	< 1% of cases	Gestational sac located within the uterine wall, completely surrounded by the myometrium and separated from the endometrial cavity
Heterotopic pregnancy	- Spontaneous conception: extremely rare - After assisted reproduction: 1–3% of patients	Concomitant presence of intrauterine and ectopic pregnancies

**Fig. 16:**
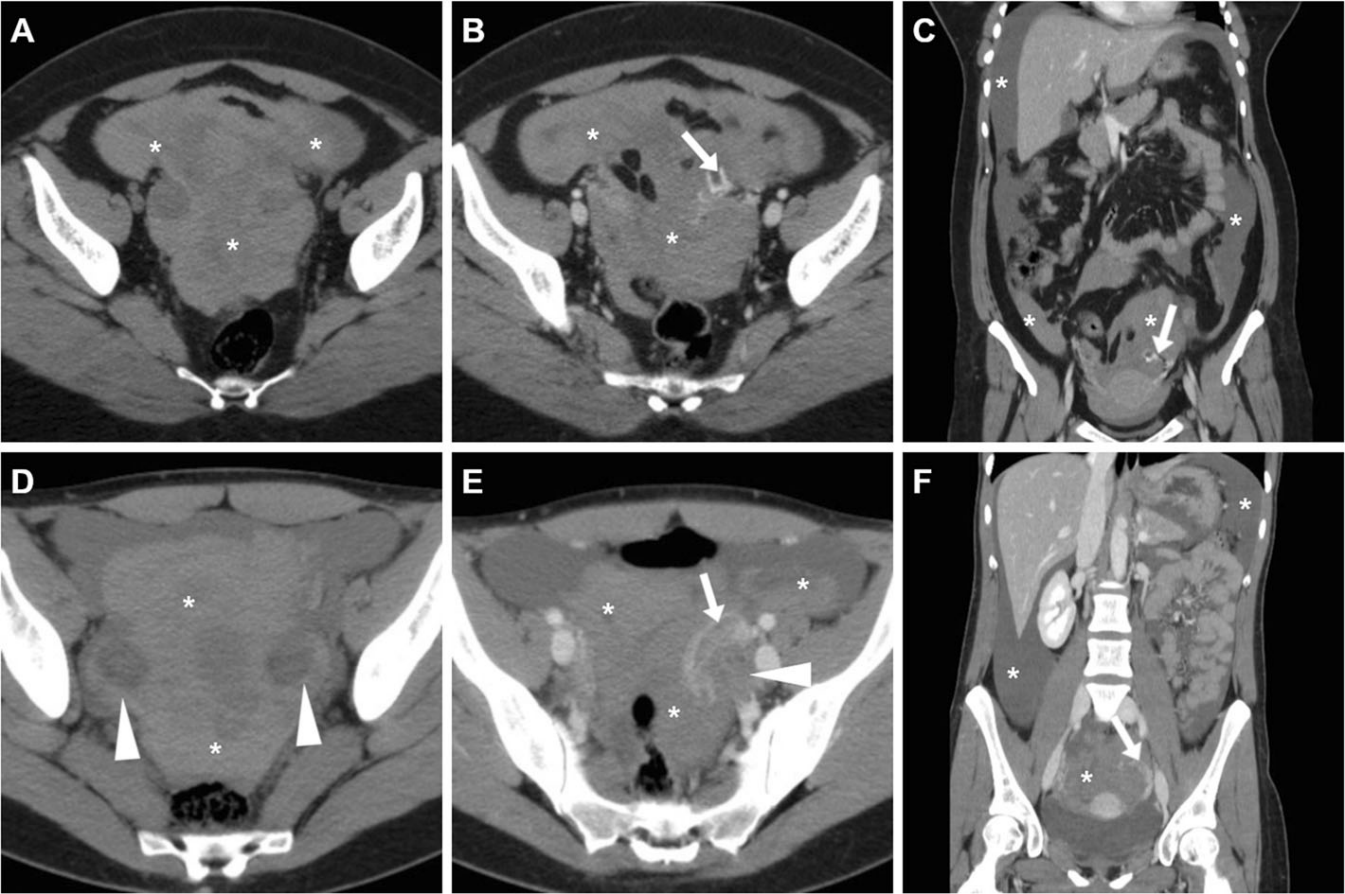
Two surgically proven cases of haemoperitoneum from early extrauterine pregnancy. **a**–**c** Unenhanced (**a**) and postcontrast (**b**, **c**) CT images show diffuse hyperattenuating peritoneal effusion (asterisk), enlarged left ovary containing a focal contrast extravasation (arrows in **b** and **c**) indicating active bleeding. **d**–**f** Unenhanced (**d**) and postcontrast (**e**, **f**) CT images show haemoperitoneum (asterisk), bilateral cystic-like ovarian lesions (arrowheads) with active haemorrhage on the left side (arrows in **e** and **f**)

The key distinction between pregnant and non-pregnant patients is determined by β-hCG levels in correlation with menstrual history. Albeit acute appendicitis is the most frequent cause of acute abdominal or pelvic pain in pregnancy, EP represents a leading consideration in patients with positive β-hCG test and non-visualisation of intrauterine pregnancy at transvaginal US. Unfortunately, US has a high specificity (near to 100%) but low sensitivity (about 15–20%) [34, 36]. Occasionally, patients with EP may undergo CT under the clinical diagnosis of severe acute abdomen or haemoperitoneum, sometimes without prior US. Alternatively, in our experience, females may be investigated after false-negative early urine pregnancy tests and are afterwards found pregnant by in-hospital β-hCG blood testing. The CT appearance of EP is an extrauterine (most usually adnexal) cystic-like structure with some degree of peripheral enhancement, representing a gestational sac, with or without haemoperitoneum (Fig. 16) [15, 37].

**Fig. 17:**
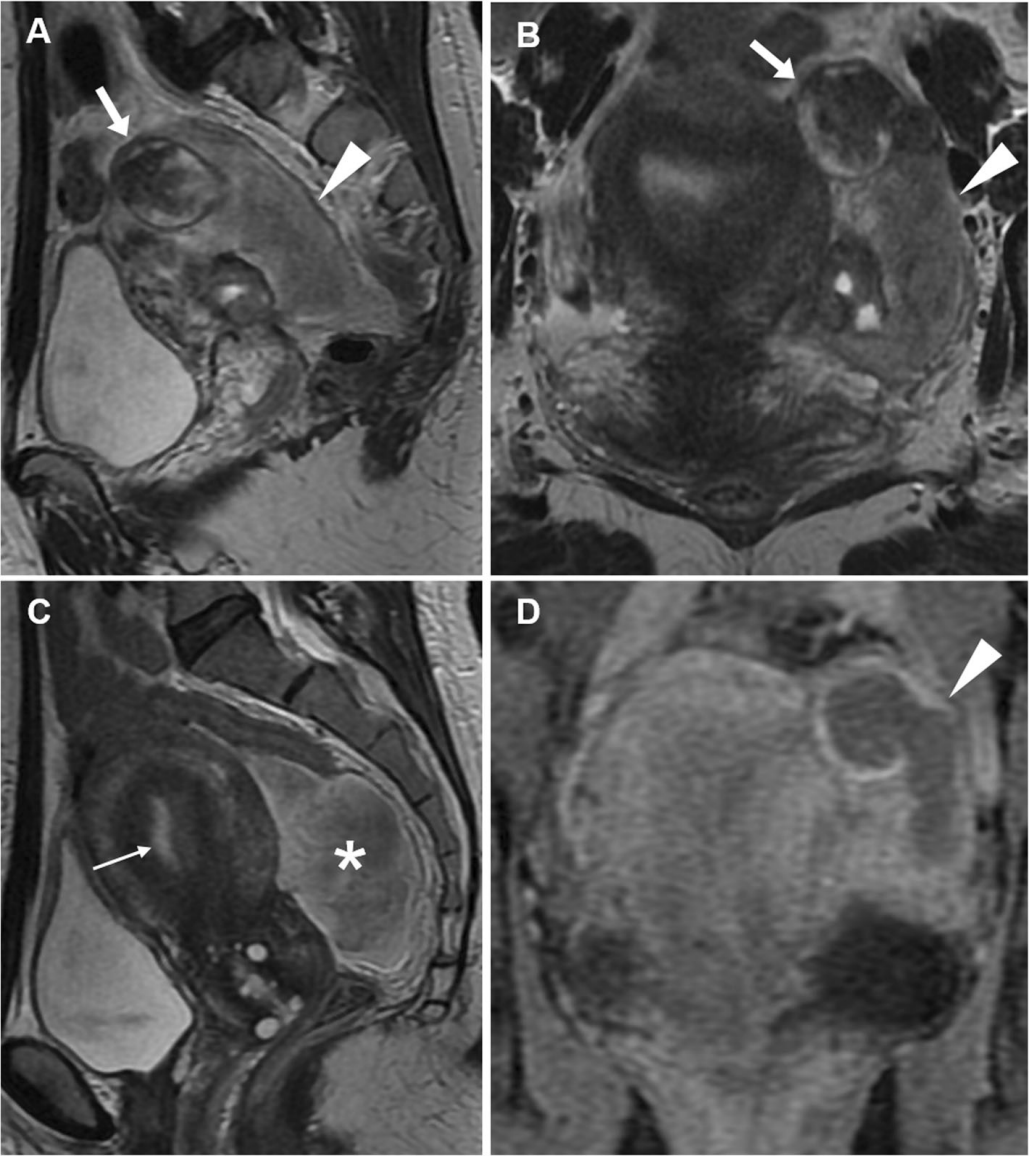
MRI of a ruptured first-trimester ectopic pregnancy in a 44-year-old woman with acute abdominal pain and elevated β-hCG levels. Sagittal (**a**) and oblique-coronal (**b**) T2-weighted images show a tubular left adnexal mass (arrowheads) containing a gestational sac in the isthmus of the fallopian tube (arrows). On oblique-coronal fat-suppressed T1-weighted image (**d**), the distended fallopian tube displays a thick hyperintense wall (arrow) consistent with haematosalpinx. Additionally, sagittal T2-weighted image (**c**) demonstrates a predominantly low-signal haemorrhagic fluid collection (asterisk) in the pouch of Douglas. Note the empty endometrial cavity (thin arrow)

In patients with equivocal clinical and sonographic findings, MRI represents an appropriate tool to make the correct diagnosis of EP. MRI findings include (a) haemoperitoneum in the pelvic cul-de-sac; (b) a heterogeneous, partly haemorrhagic, adnexal mass; and (c) tubal dilatation (haematosalpinx) with wall enhancement. The adnexal mass corresponding to the gestational sac appears as a thick-walled cystic structure which typically has high signal on T2-weighted sequences and frequently contains foci of acute blood (with variable T1-weighted signal intensity according to haemoglobin degradation stages). Unfortunately, the cystic component may not be visible, and in these cases, EP appears as a heterogeneous, mostly T2-hyperintense mass. Secondary to the invasion of the tubal wall by the trophoblasts, haematosalpinx appears as hyperintense fluid on T1-weighted sequences within the dilated fallopian tube (Fig. 17). The blood in different stages of evolution appears as iso- to hyperintense fluid on T1-weighted MR images. Additional gradient-echo T2*- and susceptibility-weighted imaging may be helpful in the identification of EP-related bloody adnexal masses. At CT and MRI, the cystic mass may show variable peripheral enhancement, corresponding to the sonographic ‘ring of fire’ sign; similarly, tubal wall enhancement has been reported, albeit the use of gadolinium contrast is controversial [9, 15, 38, 39].

Ruptured EP is still considered the primary cause of death in the first trimester of pregnancy. It is due to the invasive growth of trophoblast into the wall of the fallopian tube; the risk of rupture increases with the enlargement of the EP [40].

Imaging findings of ruptured EP include a poorly defined complex adnexal mass with the presence of pelvic fluid; the identification of bloody effusion (measuring 30–45 HU) in the Morrison pouch and in the cul-de-sac should raise concern for a ruptured EP. On contrast-enhanced images, active bleeding may be detected, especially in the case of a massive rupture. A specific CT finding is the sentinel clot sign: the presence of a hyperdense clot (> 60 HU) within the adnexum, thus confirming the source of bleeding [35, 40].

At MRI, diagnostic features of tubal rupture include the disruption of tubal wall enhancement and the occurrence of acute haematoma (low T2 signal intensity outside the implantation site). However, in pregnant patients, the detection of blood in the pelvic cul-de-sac is not specific for EP; the differential diagnosis includes haemorrhagic corpus luteum cyst and spontaneous abortion; however, the association of haemorrhagic fluid without an intrauterine pregnancy should raise suspicion for EP [9, 15, 38, 39]. Some authors reported that the size may help in the differentiation between ruptured EP (median 3.76 cm) and corpus luteum (2 cm) [15, 37].

Overall, due to its higher contrast resolution, MRI may detect EP more accurately than CT. The value of MRI in diagnosing EP has been evaluated in retrospective studies that included the presence of a direct sign (ectopic gestational sac) and indirect signs (haematosalpinx, adnexal haematoma and bloody effusion). The diagnostic accuracy of the direct sign was 92%, with a sensitivity of 91.3% and specificity of 100%, more accurate than that of any single indirect sign; accuracy increased up to 100% when diagnostic criteria required the presence of a direct sign or at least two indirect signs [41]. Furthermore, using T2*-weighted sequences in diagnosing EP sensitivity, specificity and accuracy were, respectively, 95%, 100% and 96%, due to the capability of T2*-weighted images to identify fresh haematoma [39].

Laparoscopic surgery is the gold standard treatment for EP. Indications include rupture, pain and haemodynamic instability. However, in selected patients, expectant management or pharmacotherapy (methotrexate) is being increasingly used [34, 36].

## Adnexal torsion

The rare adnexal torsion (AT) accounts for 2–3% of gynaecologic emergencies, involves partial or complete rotation of the ovarian vascular pedicle in the suspensory ligament and preferentially occurs on the right side because of contralateral protection from the sigmoid colon. It may affect the ovary, the fallopian tube or both, concomitant ovarian and tubal torsion being the most common event (up to 67% of cases) [42]. AT occurs in the first four decades of life and is uncommon in normal ovaries and in prepubertal girls with markedly mobile fallopian tubes. In reproductive age females, 50% to 90% of AT cases have an underlying ovarian mass (usually greater than 5 cm in diameter) that acts as a lead point for torsion, such as a large cyst, endometrioma, hyperstimulated ovary or benign tumour: a mature cystic teratoma represents the most common aetiology. Conversely, endometriomas and adnexal malignancies occasionally cause torsion because of fixation to adjacent structures [9, 43, 44].

The usual presentation is abrupt-onset lower abdominal pain radiating to the ipsilateral flank or groin, with adnexal tenderness. Alternatively, presentation is often nonspecific with intermittent pain, low-grade fever, nausea and vomiting. Recurrent attacks may be due to episodes of torsion and detorsion. In the early phase of AT, the low-pressure venous and lymphatic outflow is compromised, causing ovarian oedema, congestion and enlargement. If torsion persists, over time, the arterial circulation is impaired, thus resulting in ischaemia and haemorrhagic infarction. Therefore, the extent of imaging findings depends on the duration of torsion [42, 45].

In this respect, it is important to remember that the ovary has a dual blood supply:( a) from the ovarian artery (arising from the abdominal aorta), contained in the infundibulopelvic ligament that extends from the pelvic sidewall to the ovary; and (b) from the ovarian branch of the uterine artery (arising from the iliac internal artery), contained in the utero-ovarian ligament that connects the ovary to the uterus. Ovarian torsion occurs when an ovary twists on its ligamentous supports (both the infundibulopelvic ligament and the utero-ovarian ligament) [46].

US represents the primary imaging modality to assess ovarian torsion. During ovarian torsion, due to the abovementioned dual blood supply, colour Doppler US may still demonstrate some arterial vascular flow. Nevertheless, in the presence of suggestive clinical and imaging findings, this persistence of adnexal vascularisation does not rule out torsion [47].

**Fig. 18:**
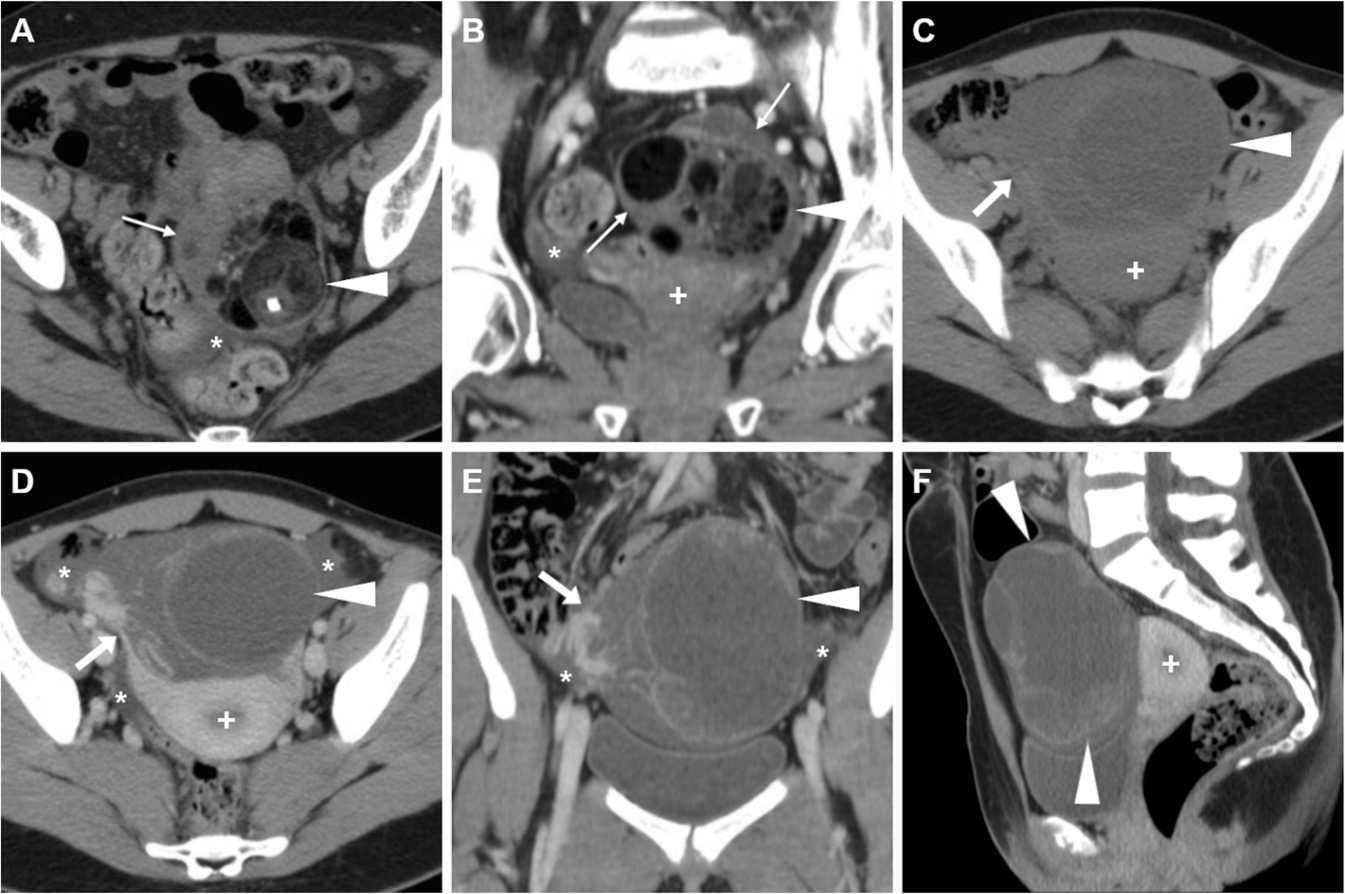
Two surgically proven cases of adnexal torsion. **a**, **b** Axial (**a**) and coronal (**b**) CT images show enlarged left ovary (arrowheads) with thickened oedematous periphery (thin arrows), containing a mixed-attenuation roundish mass with fat-attenuation foci and a calcification, corresponding to a mature cystic teratoma. Note the ipsilateral attraction of uterus (+), minimal fluid in the peritoneal cul-de sac (asterisk in **a**). **c**–**f** Unenhanced (**a**) and post-contrast (**d**–**f**) CT images show large midline pelvic mass (arrowheads) consistent with malignant teratoma with poor, irregular enhancement that displaces the uterus and bladder. Note the peritoneal effusion (asterisk) and congested ovarian vessels on the right side

After inconclusive US (lesion not clearly depicted or ambiguous findings), in premenopausal women, MRI represents the better technique to assess suspected AT, but CT is often performed in patients with alternative presumptive diagnoses. At CT, the unilaterally enlarged ovary (usually greater than 5 cm) is displaced from its expected site and often located on the midline, and the uterus is attracted towards the ipsilateral side by the shortened adnexal ligament. The oedematous ovary may show eccentric or concentric wall thickening or a ‘target-like’ pattern corresponding to peripheral displacement of follicles. Congested, enhancing ovarian blood vessels are generally seen; in this regard, it should be recalled that the pathognomonic twisted pedicle usually shows a spiral configuration, but may also present as a solid-like component adjacent to the ovarian mass. Pelvic free fluid and inflammatory fat stranding are generally present. In full-blown AT, haemorrhage appears as a hyperattenuating area and adnexal contrast enhancement is minimal or absent. When present, a mature cystic teratoma shows calcifications, foci of fat attenuation and enhancing mural nodules (Fig. 18) [9, 42, 45, 47–49].

**Fig. 19:**
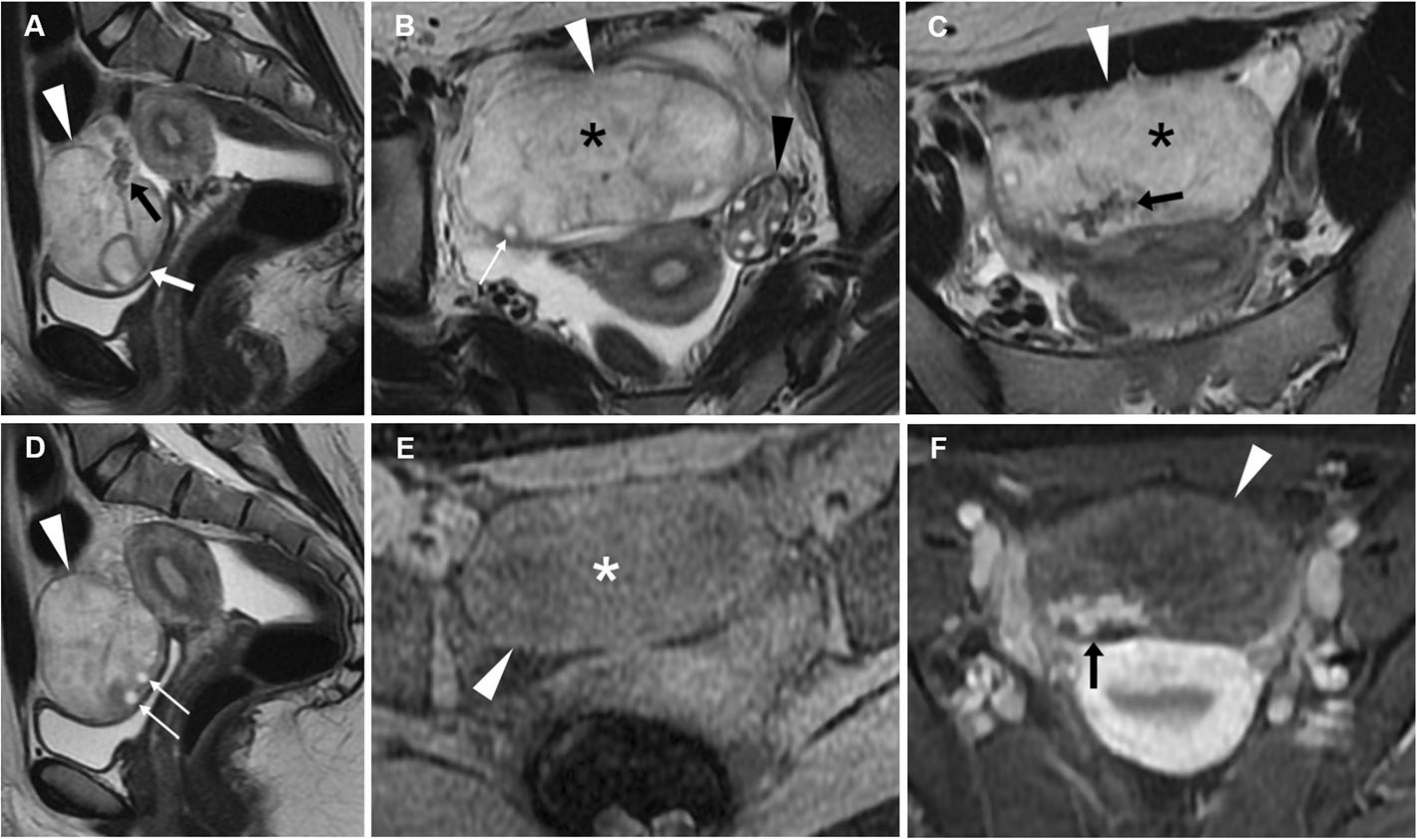
Right adnexal torsion in a 24-year-old woman with acute abdominal pain and leukocytosis. Multiplanar T2-weighted (**a**–**d**), oblique-axial precontrast fat-suppressed T1-weighted (**e**) and oblique-axial gadolinium-enhanced fat-suppressed T1-weighted (**f**) images show an enlarged (10 cm diameter) right ovary (arrowheads) with afollicular T2-hyperintense central stroma (asterisk in **b** and **c**), peripheral follicles (‘pearl string sign’, thin arrows in **b** and **d**) and corpus luteum (arrow in **a**). The normal left ovary is also seen (black arrowhead in **b**). Note the engorged blood vessels along the posterior portion of the right adnexa (black arrows in **a**, **c** and **f**). The enlarged right ovary shows homogeneous intermediate T1-weighted signal intensity (asterisk in **e**), without hyperintense haemorrhagic changes, reflecting the early stage of torsion. The patient underwent laparoscopic detorsion with adnexal sparing

Also in acute and in subacute settings, MRI may depict more clearly both direct and indirect findings of AT. The twisted pedicle, containing the thickened fallopian tube and the ligamentous support with its vascular structures, presents as a beak-like protrusion adjacent to the ovarian mass or to the enlarged ovary. In the early stage, when vascular congestion and oedematous changes prevail, the thickened (diameter > 10 mm) fallopian tube appears hypointense on T1-weighted images and hyperintense on T2-weighted images. In this stage, contrast agent administration allows to demonstrate the characteristic thickened swirling configuration of vascular structures. When ischaemic alterations and haemorrhagic infarction occur, the thickened fallopian tube may show high signal intensity on T1-weighted images. Post-contrast MRI sequences with post-processing subtraction techniques can demonstrate minimal or absent enhancement of the mass presenting high signal intensity on unenhanced T1-weighted fat-saturated images. An enlarged oedematous ovary with central afollicular stroma hyperintense on T2-weighted images and peripherally displaced follicles in a string-like appearance (‘pearl string sign’) is a quite specific sign of ovarian torsion; corpus luteum may be seen in the second half of the menstrual cycle (Fig. 19). In this early stage, ovarian stroma shows enhancement after contrast agent administration [9, 42, 45, 47–50].

At a later stage, haemorrhagic alterations develop, highly associated with infarction and secondary necrosis. Subacute haemorrhage shows high signal intensity on fat-saturated T1-weighted images and may involve the periphery of the enlarged ovary, as T1-hyperintense rim, or the central stroma. In this stage, heterogeneous, minimal or absent enhancement on gadolinium-enhanced subtraction of fat-saturated T1-weighted images confirm the evolution towards infarction [9, 42, 45, 47–50]..

Petkovska et al. described a perifollicular T2 hypointense rim correlating with perifollicular haemorrhage; the absence of this finding should be useful as a predictor of ovarian viability [51].

DWI, with both visual assessment and quantitative ADC measurement, may be useful in the diagnosis of ovarian torsion. In patients with ovarian torsion, the ADC value of the torsed ovary is significantly lower than that of the nonaffected side [52]. Kato et al. found that ADC values in swollen ovarian stroma of torsed ovaries were significantly lower in patients with haemorrhagic infarction than in those without. The cytotoxic oedema and haemorrhage that occur in ovarian torsion with haemorrhagic infarction would explain the restricted diffusion and low ADC values [53].

In patients with torsed ovarian masses, at visual assessment, higher signal intensity of the wall of the ovarian lesion on DWI significantly correlates with haemorrhagic infarction. Moreover, the high-contrast resolution of DW images may be useful to detect the twisted pedicle, reflecting in some cases of fallopian tube necrosis [54]. The contribution of DWI in the early diagnosis of ovarian torsion could avoid contrast media administration especially in some categories of patients, such as children and pregnant women [52]. Timely diagnosis of AT is crucial because the likelihood of preserving a viable ovary decreases over time. Surgical treatment should be performed within 48 h from the onset of pain to improve the outcome. Nowadays, laparoscopic detorsion is the treatment of choice in reproductive age females. In postmenopausal women and in presence of ovarian tumours, oophorectomy is required [44].

### Isolated fallopian tube torsion and paraovarian/paratubal cysts torsion

Isolated fallopian tube torsion without ovarian torsion is exceptionally rare and requires surgical intervention too. Predisposing factors are hydrosalpinx, tubal ligation, tubal or paratubal cystic and solid masses [9].

CT, better if with multiplanar reformatted images, may show an adnexal structure distinct from the ovary. MRI usually shows a distended dilated fallopian tube with thickened walls; the tube may demonstrate a vortex-like appearance (due to more twists) distant from the ipsilateral ovary, and the latter appears normal. Thick and twisted pedicle can also be observed (whirlpool sign) [19, 50].

Paraovarian or paratubal cysts are unilocular cystic structures located in the broad ligament, between the fallopian tube and the ovary. Isolated torsion of paraovarian/paratubal cysts rarely occurs and its prevalence is higher in children than in adults. MR imaging generally demonstrates a unilocular cyst distinct from the ipsilateral ovary, hyperintense on T2-weighted images and hypointense on T1-weighted images. The signal intensity of the cystic content may be slightly high on T1-weighted images due to haemorrhagic changes related to torsion [9].

### Differential diagnosis

The differential diagnosis of AT includes massive ovarian oedema and ovarian hyperstimulation syndrome (OHSS). The former entity is a rare, benign condition thought to result from intermittent or partial torsion of the mesovary compromising the venous and lymphatic drainage but with preserved arterial supply [55].

**Fig. 20:**
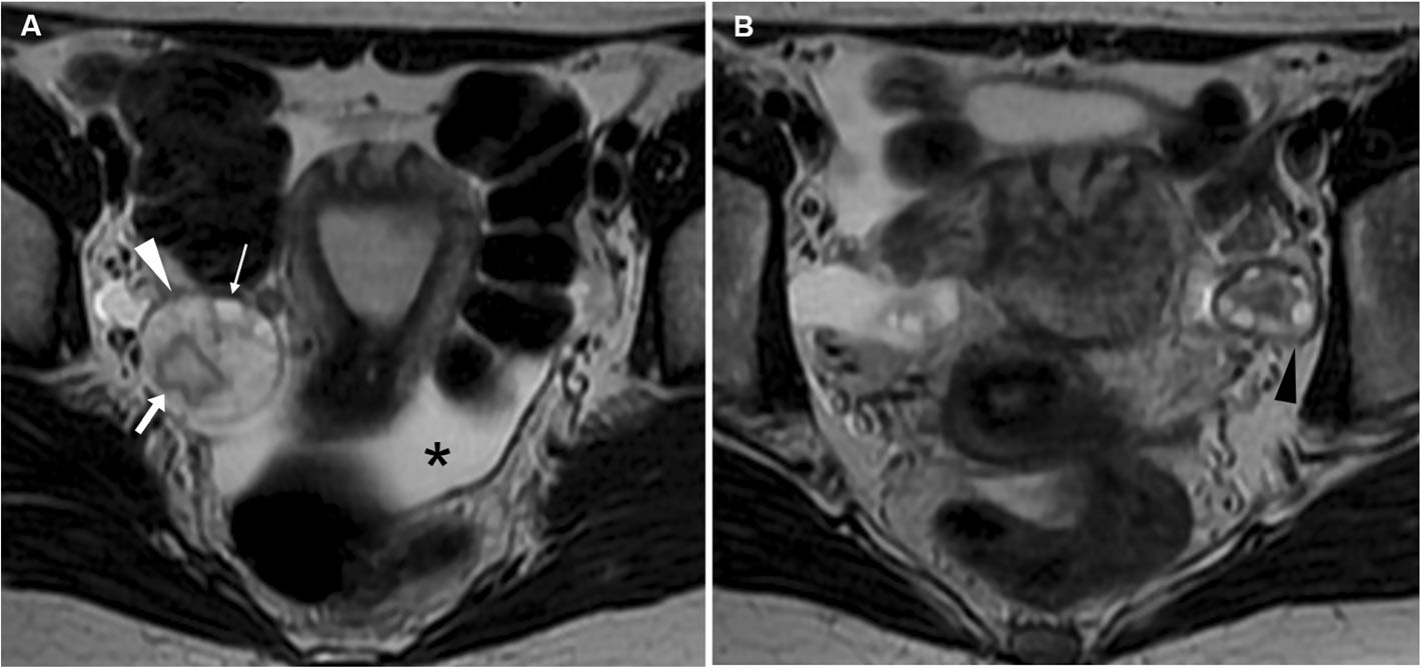
Unilateral massive ovarian oedema in a 23-year-old woman with recurrent self-limiting episodes of acute pelvic pain in the last year. Oblique coronal T2-weighted image (**a**) shows enlargement (4 cm diameter) of the right ovary (arrowhead) characterised by oedematous hyperintense stroma; follicles (thin arrow) and corpus luteum (arrow) are displaced at the periphery of the cortex. Moderate peritoneal effusion (asterisk) coexists. Oblique coronal T2-weighted image (**b**) demonstrates the normal-appearing left ovary (black arrowhead)

The imaging appearance of massive ovarian oedema is similar to that of AT: an enlarged ovary with oedematous stroma and multiple nonovulatory follicles pressed towards the periphery of the cortex (Fig. 20) [56]. However, patients’ clinical history generally records self-limiting episodes of abdominal pain of different durations. The features of pain vary (acute pain or progressive and profound diffuse pain) depending on the rapidity of the torsion; menstrual irregularities, infertility, abdominal distension, signs of virilisation and precocious puberty are also reported [57, 58].

OHSS is an iatrogenic complication of assisted reproduction techniques, characterised by bilaterally enlarged ovaries with several, peripherally located cysts accompanied by fluid shift from the intravascular to third space from increased capillary permeability that may cause the development of ascites, pleural effusions and oliguria in severe forms [59]. Typical imaging findings at CT and MRI include bilateral symmetric enlargement of ovaries due to the presence of multiple cysts representing enlarged follicles or corpus luteum cysts. Cystic content can be fluid-like or haemorrhagic; in the latter case, cysts demonstrate higher attenuation at CT and high T1 signal intensity at MRI. The characteristic peripheral location of the follicles, separated by thin septa and surrounding a central core of ovarian stroma, has been referred to as ‘wheel spoke’ appearance [60]. Differently from cystic neoplasms, OHSS lacks abnormal enhancing solid components within the cystic lesions; in addition, follow-up imaging should demonstrate resolution. It should also be remembered that enlarged hyperstimulated ovaries are themselves at risk for torsion [61]. If torsion occurs in hyperstimulated ovaries, the latter may demonstrate asymmetrical augmentation, abnormal enhancement, twisted pedicle and eventually haemorrhage [50].

Other features of OHSS include free fluid in the peritoneal, pleural and pericardial cavity; the free fluid seen in OHSS is generally simple ascites, although it may display slightly higher attenuation due to ruptured haemorrhagic cysts. The modified Golan classification categorises OHSS as mild, moderate or severe on the basis of symptoms severity and radiologic findings [62].

### Pelvic congestion syndrome

Congested pelvic veins without AT are the hallmark of the pelvic congestion syndrome (PCS), an underdiagnosed condition that affects 9% of premenopausal women and may be exacerbated by intra-abdominal pressure. The multifactorial pathogenesis involves a family history of varicose veins, previous pelvic surgery, multiple pregnancies, hormonal influences and retroverted uterus. Although the key symptom is chronic dull pelvic pain that worsens in the premenstrual phase, acute and severe pain may also occur. Flow reduction, thrombosis and mass effect on nerves may lead to worsening pelvic pain. Other clinical manifestations may include dyspareunia, postcoital ache, dysmenorrhoea and perineal pain [63, 64].

Similarly to male varicocele, dilatation of the gonadal veins and pelvic venous plexus more commonly occurs on the left side because of left ovarian vein draining into left renal vein before reaching the inferior vena cava. Mechanical extrinsic compression of drainage veins such as in retroaortic left renal vein, in May-Thurner syndrome (left common iliac vein compression against the lumbar spine by the right common iliac artery) or in nutcracker syndrome (left renal vein compression between superior mesenteric artery and aorta), may lead to venous congestion and engorgement of the ovarian vein [65]. In this latter syndrome, the abrupt narrowing, with a triangular shape, of the proximally dilated left renal vein between the abdominal aorta and the superior mesenteric artery is referred to as the ‘beak sign’. It is one of the most useful CT findings for the diagnosis of nutcracker syndrome, with a sensitivity of 91.7% and a specificity of 88.9% according to the literature [66, 67]. MR angiography provides findings similar to those of CT with the advantage of being less invasive and without radiation exposure in comparison with retrograde venography [68].

**Fig. 21:**
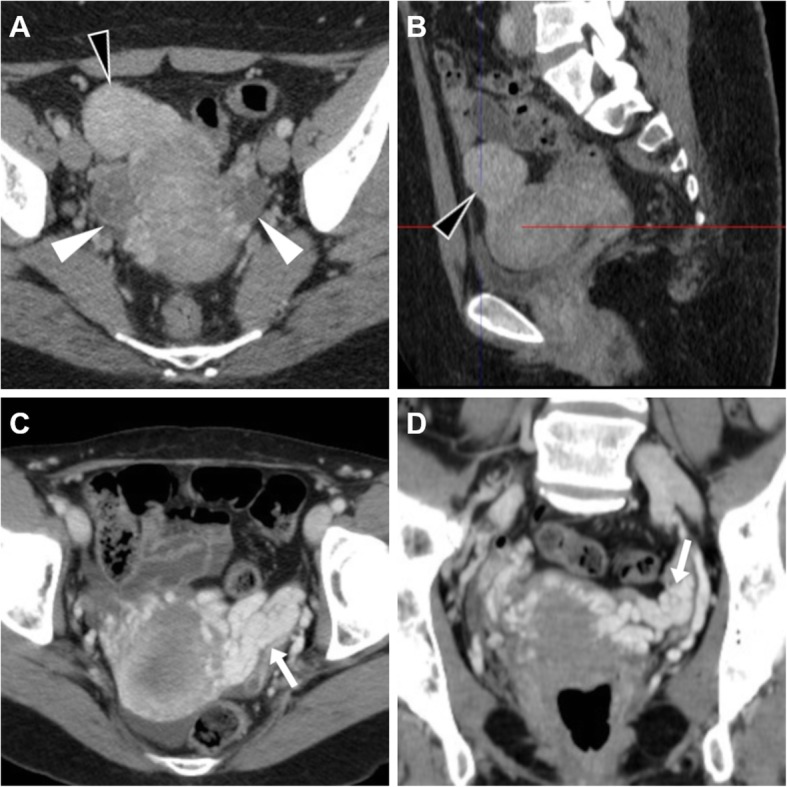
Pelvic congestion syndrome. Axial (**a**) and coronal (**b**) CT images showing asymmetric dilatation of the pelvic venous plexus (arrows) reaching 6 mm maximum calibre on the left side

**Fig. 22:**
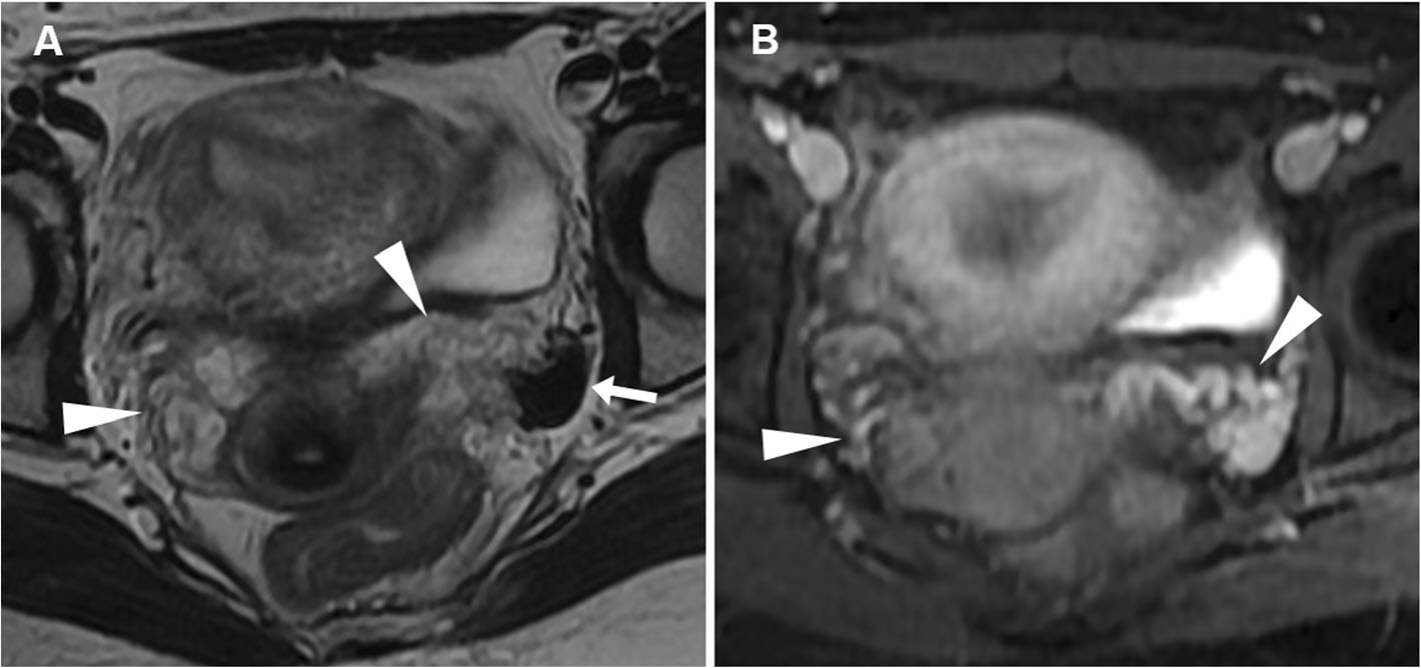
MRI of pelvic congestion syndrome in a 26-year-old woman with chronic pelvic pain and a recent severe exacerbation. Oblique-coronal T2-weighted image (**a**) shows ectatic vascular structures in the parametria, showing predominantly high signal intensity (arrowheads); in the left parametrium, some varices (arrow) display low signal intensity. Corresponding oblique-coronal gadolinium-enhanced fat-suppressed T1-weighted image (**b**) demonstrates vascular enhancement of the abovementioned structures (arrowheads), thus confirming pelvic varices

**Fig. 23:**
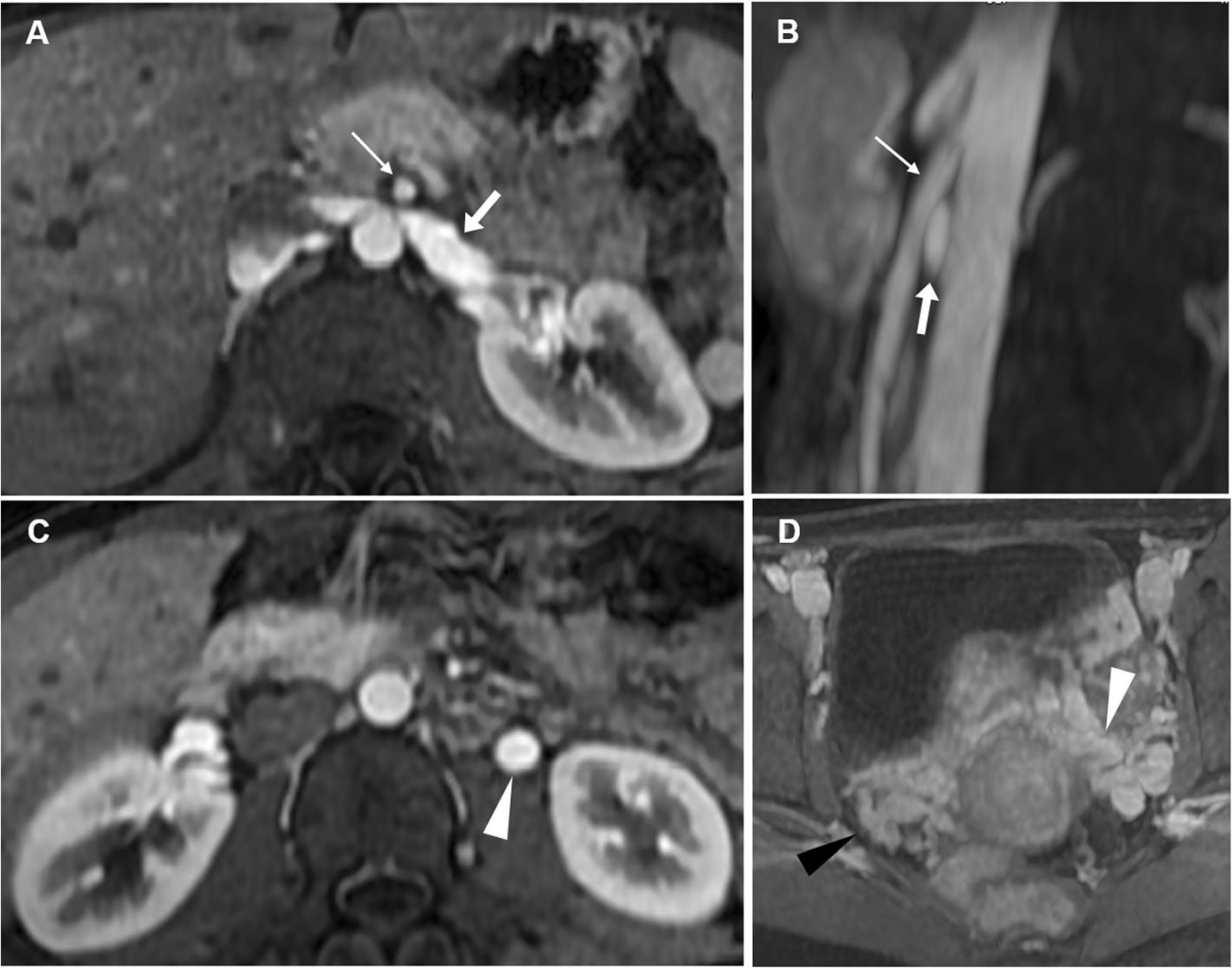
Pelvic varices from nutcracker syndrome in a 24-year-old woman with severe pelvic pain. Axial gadolinium-enhanced fat-suppressed T1-weighted images (**a**, **c**) demonstrate compression of left renal vein (‘beak sign’, arrow) between the abdominal aorta and superior mesenteric artery (thin arrow) and enlarged left gonadal vein (arrowhead). Additional sagittal maximum-intensity projection (MIP) reconstruction (**b**) demonstrates the left renal vein (arrow) within a narrow angle between the abdominal aorta and the superior mesenteric artery (thin arrow), consistent with ‘nutcracker syndrome’. Axial angiographic MIP image (**d**) demonstrates dilated, engorged left parametrial and uterine veins (arrowhead). Note the dilatation of contralateral parametrial plexus (black arrowhead) due to the anastomosis via the arcuate uterine veins

At CT, tortuous and dilated (> 4 mm) tubular structures nearby the ovary and uterus show slow blood flow and enhancement synchronous with veins. The diagnostic criteria for PCS require at least four ipsilateral parauterine veins of varying calibre, at least one measuring more than 4 mm in diameter, or an ovarian vein diameter greater than 8 mm (Fig. 21). Similarly, MRI depicts pelvic varices as multiple dilated vessels lying on uterus (and within the myometrium such as arcuate veins), ovaries and pelvic sidewall that can extend inferiorly to communicate with paravaginal and thigh venous plexus [64, 69–71]. Dilated veins are hyperintense on gradient-echo T1-weighted images; on T2-weighted MR images, they may show no signal intensity due to flow-void artefacts or mixed low and high signal intensity because of the relatively slow flow inside the vessels. Gradient-echo fat-suppressed T1-weighted sequences after intravenous administration of contrast material demonstrate vascular enhancement of pelvic varices, thus confirming the diagnosis (Figs. 22 and 23) [64]. Asciutto et al. reported MRI to have levels of sensitivity and specificity for PGS respectively of 88% and 67% for ovarian veins, 100% and 38% for iliac veins and 91 and 42% for pelvic plexus [72].

**Fig. 24:**
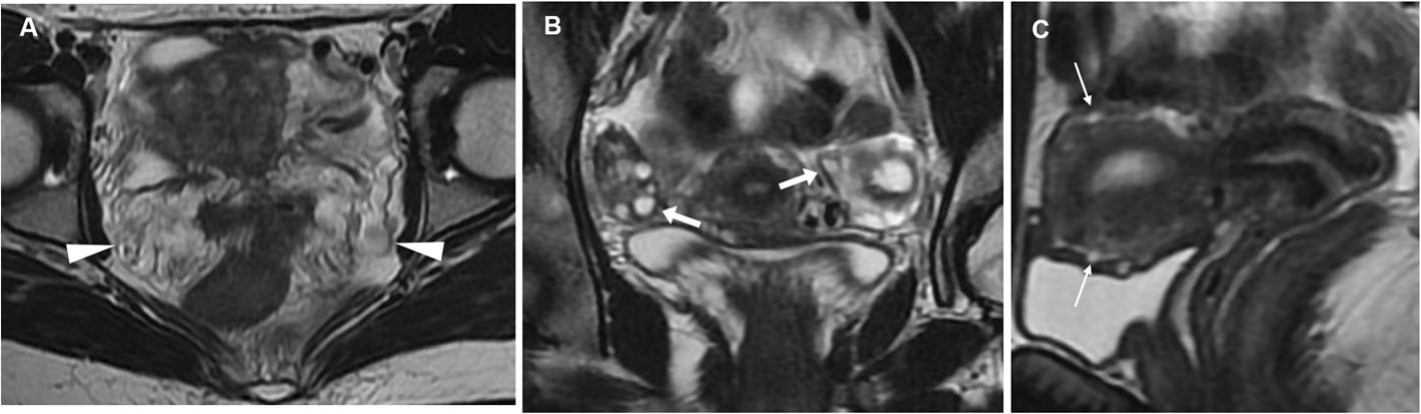
In the same patient as in Fig. 22, multiplanar T2-weighted images demonstrate severely congested pelvic veins (arrowheads in **a**), cystic components in both ovaries (arrows in **b**), and dilated arcuate veins in the subserosal portion of the myometrium (thin arrows in **c**)

Additionally, up to 50% of women with PVI present cystic formations in the ovary, ranging from scant cysts to classic polycystic ovary aspect (Fig. 24) [64]. The mechanism underlying these cystic changes in the ovary is not completely known, although a relationship with increased oestrogen levels has been reported [73].

## Conclusion

Although liberal use of CT should be discouraged in childbearing age women, radiologists performing urgent CT studies may encounter an unsuspected acute female genital disorder that warrants immediate gynaecologic examination, further workup or prompt intervention. Therefore, familiarity with these conditions and their imaging appearances is crucial to avoid missing or misinterpreting clinically important entities which may require surgical treatment. If clinical conditions and scanner availability allow, MRI is superior to CT for further characterisation of acute disorders such as adnexal torsion, corpus luteum and haemorrhagic cysts [8, 22].

The following second instalment will present acute uterine disorders causing abnormal vaginal bleeding in non-pregnant women (including endometrial polyps, complicated leiomyomas and uterine inversion) and the imaging spectrum of PID and atypical genital infections.

## Data Availability

Data sharing is not applicable to this article as no new data were created or analysed in this study.

## Abbreviations

ADC: Apparent diffusion coefficient
ASSET: Array spatial sensitivity technique
AT: Adnexal torsion
CT: Computed tomography
DWI: Diffusion-weighted imaging
ED: Emergency department
EP: Ectopic pregnancy
ESUR: European Society of Urogenital Radiology
HU: Hounsfield units
MARS: Metal artefact reduction sequences
MRI: Magnetic resonance imaging
OHSS: Ovarian hyperstimulation syndrome
PCS: Pelvic congestion syndrome
PID: Pelvic inflammatory disease
STIR: Short tau inversion recovery
US: Ultrasound
β-hCG: Beta human chorionic gonadotropin
